# A symphony of functioning: exploring the interplay of cognition, movement, and visual demands in adolescents on the autism spectrum using mobile brain-body imaging (MoBI)

**DOI:** 10.1186/s11689-026-09677-1

**Published:** 2026-02-10

**Authors:** Paige R. Nicklas, Lisa N. Cruz, Carol Terilli, Erin K. Bojanek, Pierfilippo De Sanctis, Edward G. Freedman, Sophie Molholm, John J. Foxe

**Affiliations:** 1 The Frederick J. and Marion A. Schindler Cognitive Neurophysiology Laboratory, Department of Neuroscience, School of Medicine and Dentistry, The Del Monte Institute for Neuroscience, University of Rochester, Rochester, NY 14642 USA; 2 The Golisano Intellectual and Developmental Disabilities Institute, Department of Neuroscience, University of Rochester School of Medicine and Dentistry, Rochester, NY 14642 USA; 3 The Cognitive Neurophysiology Laboratory, The Rose F. Kennedy Intellectual and Developmental Disabilities Research Center, Departments of Pediatrics & Neuroscience, Albert Einstein College of Medicine, Bronx, NY 10461 USA; 4 Department of Psychiatry & Behavioral Sciences, Stanford University School of Medicine, Palo Alto, CA USA; 5 Department of Neurology, Division of Cognitive and Motor Aging, Albert Einstein College of Medicine, Bronx, NY USA

## Abstract

**Background:**

Autism Spectrum Disorder (ASD) is characterized by differences across multiple intertwined functional domains, including cognitive, sensory, and motor processes. There is a need to understand how concurrent demands in different domains can impact performances in one another, as the simultaneous processing and execution of tasks from different domains is how most normal daily tasks and activities are completed. Differences in integration are thought to underly many characteristics of ASD, and therefore understanding how the brain processes multi-modal demands in both typically and neurodivergently developing populations is vital, and revealing how neural activity adapts to meet multi-modal demands can serve as valuable markers for supporting diagnosis and treatment decisions.”

**Methods:**

We used Mobile Brain-Body Imaging (MoBI) to simultaneously record 64 channel electroencephalography (EEG), motion-tracking, and response inhibition task performance in adolescents (ages 13–23, mean 16.96 years) who have ASD and typically developing (TD) counterparts. We designed experimental conditions that either did or did not include a motor demand (standing or treadmill walking), sensory demand (static field or optical flow), and cognitive demand (completing task or not) to investigate single, dual, and tri-modal impacts on ERPs, gait kinematics, and task accuracy and speed.

**Results:**

The TD group was significantly more accurate when walking. The ASD group did not increase task accuracy despite making similar adjustments to response speed when going from standing to walking. Optic flow did not impact task accuracy or for either group but did impact response speed. Similarly, walking impacted N200 and P300 amplitudes and latencies, but the addition of visual flow did not further these impacts. The ASD group’s neural activity showed differences that were similar in direction but weaker in magnitude to the addition of more demands (walking and flow), compared to the TD group.

**Conclusions:**

There is a complex interplay between motor, cognitive, and sensory functions and we provide evidence here that cross-domain integration of these in adolescents is different in ASD than those who are typically developing. These results suggest that coupled neural and gait responses during multi-modal demands may serve as a potential marker of altered cross-domain integration in adolescents with ASD. Future research should further investigate these relationships with multi-modal methods like MoBI.

**Supplementary Information:**

The online version contains supplementary material available at 10.1186/s11689-026-09677-1.

## Introduction

Everyday life necessitates processing, evaluating, and executing demands across multiple domains: cognitive, motor, and sensory. The ability to do so is vital to typical, healthy functioning. These domains of functioning are not mutually exclusive, which necessitates the study of these domains in tandem to shape a quantifiably holistic understanding of their interplay in neurotypical and neurodivergent populations. One common way to investigate the impact of demands of multiple domains on each other and their outputs is “dual-task” experimental designs. This involves comparing performance on a single-domain task alone (single-task) to performance on the same task when a simultaneous demand of a different domain is introduced, such as walking (dual-task) [1, 2]. The deficits in chosen metrics of task performance observed when comparing dual-tasking (DT) to single-tasking (ST) are referred to as “dual-task costs”, and are representative of the adaptations required to successfully complete both tasks.

Historically, dual-task studies have not been able to simultaneously access the neural and kinematic underpinnings of the phenomena they were investigating. However, with technological and computational advances, methods have emerged in the past decade that allow researchers to capture these phenomena across domains, one of which is “Mobile Brain/Body Imaging” (MoBI). MoBI experiments involve simultaneous, sub-millisecond recording of three data streams: (1) kinematics via motion capture, (2) neurophysiology via EEG, and (3) behavior via task performance. By doing so, investigators can create more naturalistic experimental environments, and begin to characterize function under more “real-world” conditions. MoBI has been shown to have good signal-to-noise ratios for the event-related potentials (ERPs) collected during walking recordings [3, 4], long-term test-retest stability [5], and accurate EEG electrode localization capabilities [6]. This allows for insight into neurophysiological mechanisms underlying processes during naturalistic, multi-domain behaving [3, 4, 6]. 

There are limits to the processing demands that can be made before behavioral and/or motoric performance declines. Studies using dual-task paradigms can investigate the flexibility of neural processing and the extent to which these changes can stave off performance limits. MoBI studies can also identify changes in this threshold due to developmental disability, healthy aging or neural degeneration resulting from disease [7–12]. One challenge of characterizing multi-modal dynamics is that the measurable impacts of dual-tasking can vary widely. A 2023 study by Patelaki et al. [13] reported that over half of their healthy neurotypical young adult participants actually improved on both cognitive task and gait performance during dual task versus single task conditions (i.e., while dual tasking) [13]. This provides further evidence of variations in the extent of impacts of dual-task paradigms, and shows that some individuals have more effective neural flexibility to use those resources. Further, dual-task costs are greater when the cognitive task requires inhibition, compared to working memory tasks [14], indicating tasks of response inhibition are a good choice for robustly introducing competition for neural resources.

To characterize diagnosis-specific differences, dual-tasking has been investigated in several clinical populations and have been shown to be greater, compared to healthy controls, in individuals with multiple sclerosis [7], Parkinson’s disease [15, 16], traumatic brain injury [17–19], older adults with mild cognitive impairment [11, 20], and those who have suffered a stroke [21–23], though this is not an exhaustive list. One study found specific, distinct cognitive-motor profiles in individuals with Williams syndrome and Down syndrome during dual-tasking [24]. For neurodevelopmental disorders specifically, individuals often have motor atypicalities [25] and greater difficulty when multi-tasking [26, 27]. Using MoBI with dual-tasking can help elucidate signatures of the stages of processing and respective neural and/or locomotive processes of dysfunction, and has been used in various ages and populations to assess cognitive-motor interactions and expand understanding of the neural underpinnings of dual-task effects [3–5, 8, 11, 13, 28–30]. 

However, typically developing children under 18 years old have not yet been studied using MoBI, nor have individuals with neurodevelopmental differences. Autism Spectrum Disorder (ASD), a neurodevelopmental disorder diagnosed by differences in social communication abilities as well as the presence of restricted and repetitive behaviors, is characterized by differences in all three domains: cognition, motor, and sensory, in varying degrees of severity and combinations depending on the individual [31–33]. Therefore, using MoBI to study dual-tasking and multi-modal functioning in ASD has potential to be particularly informative in elucidating the interplay of these three domains in a more realistic experimental setup.

Cognitively, individuals with ASD have known differences across executive functions [34]. Specifically, inhibitory control deficits are commonly reported in ASD compared to their typically developing counterparts [35–37]. These differences are thought to contribute to the characteristic repetitive and stereotyped behaviors and/or restricted interests which are prevalent in, and needed to diagnose, ASD [38, 39]. 

Motor parameters from kinematic data can include walking speed, gait variability, center-of-mass displacements, and step and stride lengths [18, 40–42]. Those with ASD typically have delays in fine and gross motor development [43]. When walking, a variety of kinematic differences have been shown between those with ASD and neurotypical controls in both children [44–47] and adults [48–51], including poorer postural control [42, 46, 52, 53], increased step width [54] and higher variability of steps and strides [47, 55], reduced waking speed [51], and shorter strides [55]. Overall, gait features of individuals with ASD tend to be slower, more variable and arrhythmic than neurotypical individuals. Taken together, this raises the question about the interaction of the underlying motor and cognitive circuits during dual-tasking in ASD, and how they differ from their TD counterparts. If motoric control of walking is more taxing for available neural resources in ASD as a single-modal demand, then one may expect the addition of a cognitive task to cause greater interference in individuals with ASD than those who are typically developing.

The sensory domain also serves as part of the *Diagnostic and Statistical Manual of Mental Disorders*,* 5th Edition* (DSM-5) diagnostic criteria for ASD [38], with the majority of individuals with an ASD diagnosis having some presentation of symptoms in this domain [56, 57]. During walking, there is a continuous influx of visual information about the environment, heading direction and speed, potential obstacles, making motor and visual functioning tightly entwined [9, 46]. Adaptations and corrections needed to incorporate this information in order to navigate the environment have been shown to impact neurophysiology [10, 58, 59]. Specifically, for visual processing in ASD, the literature is mixed with some studies showing intact motion perception abilities in ASD [60, 61] that are sometimes accompanied by altered brain responses [62], but other studies showing impaired abilities in ASD [63–66]. Their posture and balance seem to be more impacted by optic flow [46, 67] and dual-tasking [68], though these studies are few.

Locomotive, cognitive, and sensory processing are interconnected. These functional domains are atypical in many individuals with ASD, and it has been proposed that motor, cognitive and sensory processing challenges, alone or in tandem, contribute to the social and communication differences in ASD [36, 53, 69–76]. Therefore, there is a significant need to use multi-modal experimental tools and paradigms capable of assessing the dynamic interplay of these domains, like MoBI, in ASD. The current study manipulated all three domains: motor, cognitive and sensory, in order to assess their individual and combinatorial impact on typically developing adolescents, and compare these in age and cognitive function matched individuals with ASD. Our experiments investigated how known inhibitory-based differences vary across motion and sensory conditions, examining potential interactions and implications for neural processing in more naturalistic contexts. To address this, we used Mobile Brain-Body Imaging (‘MoBI’). EEG event-related potentials (ERPs) of interest are those known to be elicited under similar experimental conditions: the P200, a fronto-central potential related to early attentional processing that peaks 150–300 ms after stimulus presentation; [30, 77–79] the N200 (‘N2’), a negative voltage deflection fronto-centrally located on the scalp, peaks about 200–350 ms after the stimulus [80], and is associated cognitively with stimulus distinction and conflict monitoring, and anatomically with the anterior cingulate cortex [81–83]; the P300 (‘P3’), which reflects the decision-making needed to complete the assigned task [28, 84–86], typically seen roughly 150 ms after the N2, is a positive voltage deflection located more centro-parietally and peaks 350–600 ms after stimulus onset [79]. It is understood to have a broader distribution of underlying neurological generators, likely because it’s believed to reflect parallel processing of both cognitive and motor domains of complex processes like stimulus evaluation and memory updating [28, 87, 88]. 

Neurophysiologically, the few studies in ASD which combined a response inhibition task with EEG have generally reported less robust elicitation of the associated brain responses than those observed in typically developing individuals [89–92], though the magnitude of these results are not consistent across studies, warranting further investigation. One study reported no difference between the P3 and N2 between TD and ASD adolescents, but reduced P2 amplitudes and increased latencies [93]. One group demonstrated reduced N2 amplitudes in children with ASD compared to a group of TD children [90], where another saw no difference [89], and others have seen increased N2 amplitudes in ASD [76, 94]. Given these mixed and sparse findings in the ASD literature, we focus on these ERPs to help addresses these gaps because of their established effects and functional significance in the MoBI literature [3–5, 7, 8, 13, 28, 95]. 

Due to the sample size here, we want to highlight that this study should be considered exploratory in nature. Our core thesis was that changes in gait kinematics, modulations of ERP peak amplitude and latency, and behavioral performance observed when comparing single-modal to multi-modal demands would be similar in direction, but greater in magnitude for the ASD group compared to the TD group. This would be indicative of higher multi-modal costs, and disrupted integration and execution of processes across the three domains. Further, knowledge of the sources and timing differences of multi-modal processing would inform how multi-modal information processing presents differently in ASD. Differences in timing and amplitudes of ERP components could help specify which processing stages are different in ASD. Delayed timing could indicate reduced flexibility in cognitive control processes and difficulty to adapt to multi-modal demands [11, 13, 28]. It is also possible to see recruitment of more frontal areas of the brain when multi-tasking, which has been reported in neurotypical young adults [4, 28]. A shift in neural source like this could indicate that the processing strategy itself is different between groups across different types and combinations of multi-modal demands. Results found could provide kinematic and neurophysiological markers of atypical functioning in ASD that cannot be detected by any single-modal methods or measurements, providing a more all-inclusive way to identify deficits and monitor treatment and therapy impacts.

## Methods and materials

### Participants

Participants were recruited via the Human Clinical Phenotyping Core (HCP) at the Albert Einstein College of Medicine. The HCP is part of the Rose F. Kennedy Intellectual and Developmental Disabilities Research Center (IDDRC) at Einstein, and is funded by the National Institute of Child Health and Human Development (NICHD). Participants included here were recruited through the HCP for a primary study titled “The Neurophysiological Underpinnings of Sensory-Motor Dysfunctions in Autism Spectrum Disorder.”

This current cohort includes 20 individuals with a diagnosis of ASD, and 18 typically developing individuals. In the ASD group, the average age is 16.56 years (± 2.69), and 3 participants are female. The TD group has an average age of 17.19 years (± 2.51), with 7 females. Individuals were recruited for, consented into, and participated in the study according to procedures approved by The Institutional Review Board of the Albert Einstein College of Medicine. Participants aged 18 years and older provided written informed consent. Children under 18 years provided written informed assent, accompanied by their legal guardian’s written consent. ASD diagnosis was confirmed by a clinical psychologist with expertise in the diagnosis of ASDs using the Autism Diagnostic Interview-R (ADI-R) [96], Autism Diagnostic Observation Schedule (ADOS) [97], and professional, clinical judgment. In the TD group, individuals were excluded based on history of psychiatric conditions, special education, or any other developmental issues as reported on pre-screening questionnaires, such as learning disabilities, identified syndromic causes of ASD (e.g., tuberous sclerosis, Down syndrome, Fragile X), or a history of a developmental disorder in a first-degree relative. All study procedures were compliant with the principles laid out in the Declaration of Helsinki for the responsible conduct of research.

### Experimental design & procedures

**Fig. 1:**
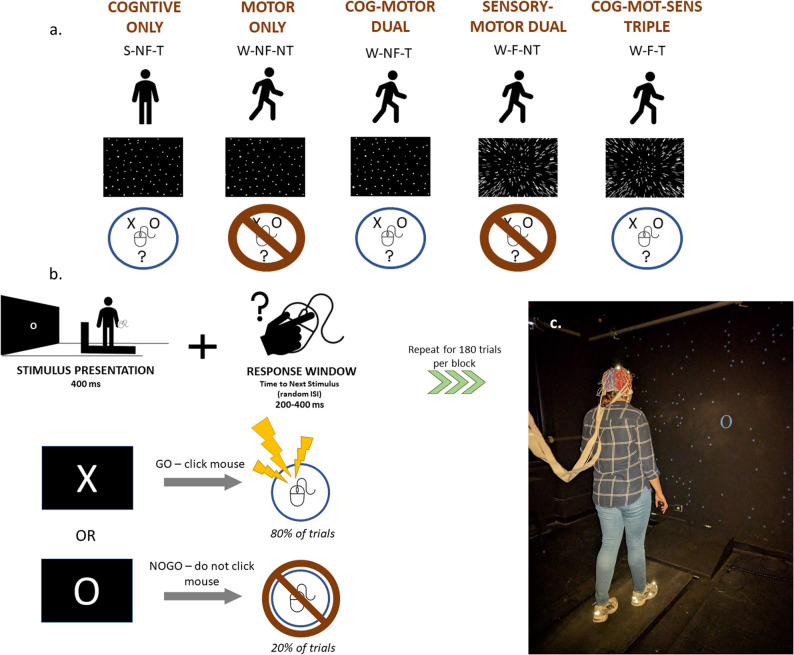
**A** Schematic of the 5 different experimental conditions. During a single block a participant was either standing (S) or walking (W) on treadmill (Motor), while performing (T) or not performing (NT) a visual cognitive task (Cognitive), with (F) or without (NF) optical flow in their visual field (Sensory). **B** Schematic of Go-NoGo response inhibition task. **C**. Photo of mock experimental set-up, not including the safety harness. Originally published in Malcolm et al., [98]

The study included 2 sessions, one included the MoBI recording session and the other for assessments. Participants were paid $15 USD an hour for their time. During the MoBI recording session, participants completed blocks containing combinations of sensory, cognitive, and motor loads. During a single block a participant was either standing (S) or walking (W) on a treadmill, while performing (T) or not performing (NT) a visual cognitive task, with (F) or without (NF) optical flow in their visual field. These three loads were combined to create the following five experimental conditions (Fig. 1a):

- S-NF-T (cognitive only)
- W-NF-NT (motor only)
- W-F-NT (motor-sensory dual-modal)
- W-NF-T (motor-cognitive dual-modal)
- W-F-T (motor-sensory-cognitive tri-modal)

Time-synchronized stimulus triggers for the cognitive task, behavioral responses, EEG, and motion-tracking were collected using Lab Streaming Layer (LSL) software (Swartz Center for Computational Neuroscience, University of California, San Diego, CA; https://github.com/sccn/labstreaminglayer). Further detail on each data stream is provided in the following methods sections.

### Cognitive task, stimuli, and behavioral performance

Participants performed a visual Go-NoGo (vGNG) response inhibition task. Participants were presented with a series of stimuli on the screen in front of them consisting of ‘X’s and ‘O’s (Fig. 1). They held a wireless computer mouse in their right hand and were instructed to press the button if the ‘X’ was displayed (Go trials), but to withhold the button press if an ‘O’ was displayed (NoGo trials). NoGo trials occurred 20% of the time. A training block was completed before beginning the experimental blocks, to ensure participant understanding of task instructions. Participants completed a maximum of 15 experimental blocks with each block having 180 trials.

Experimental conditions were presented in a pseudorandom order, such that no more than 3 walking inclusive blocks could be done in a row:

- 3 blocks of the vGNG task were performed while standing, without optical flow (S-NF-T)
- 6 blocks of the vGNG task were performed while walking on the treadmill
  - ◦ 3 of which included optical flow (W-F-T)
  - ◦ 3 blocks without optical flow (W-NF-T)
- 6 blocks where no task was performed while walking on the treadmill
  - ◦ 3 blocks with optical flow (W-F-NT)
  - ◦ 3 blocks without optical flow (W-NF-NT)

Due to the length of the experimental session, standing blocks with optical flow and performing the vGNG task, what would have been an S-F-T condition, were not included in the study design in order to decrease participant burden. This limits some statistical analyses detailed in “Statistical Analyses” section.

Visual stimuli were programmed with Presentation software version 21.1 (Neurobehavioral Systems, Albany, CA, USA) and projected (InFocus XS1 DLP, 1024 × 768 pxl) centrally onto a black wall approximately 1.5 m in front of the treadmill (LifeFitness TR-9000). Each image was presented for a duration of 400 ms. A random inter-stimulus interval (ISI) ranged from 200 to 400 ms. Images subtended 28° horizontally by 28° vertically, on average. Participants were instructed to perform the Go/No-Go task as quickly and accurately as possible (Fig. 1b). During the no-task (NT) blocks, the go/no-go stimuli were shown but participants were instructed not to respond or cognitively engage in the task. These task parameters elicit four possible response types: Hits, when an ‘X’ is displayed and the button is correctly pressed, Misses, when an ‘X’ is displayed and the button is incorrectly not pressed, False Alarms (FAs), when an ‘O’ is displayed but the button is incorrectly pressed, and Correct Rejections (CRs), when an ‘O’ is displayed and the button is correctly not pressed.

### Optic flow

Optic flow was replicated as originally presented in Malcolm et al., [98]. In all experimental conditions, task stimuli were presented on a visual field that included 200 randomly located white dots projected onto the black background, subtended on average 100 degrees vertically and horizontally. During visual optic flow conditions (W-F-NT and W-F-T), the dots radiated steadily outward from a central point of expansion. In S-NF-T, W-NF-T, and W-NF-NT conditions, the dots remained static.

### Motion-tracking & gait kinematics

Three markers were placed on each foot, one at the calcaneus and one at each 2nd and 5th distal metatarsal, for a total of 6 markers on each participant. The trajectories of the markers were recorded by the OptiTrack R2 system, an optical 3D motion analysis system (OptiTrack, NaturalPoint Inc., Corvallis, OR, United States). The set-up includes 9-cameras that electronically connect to ARENA v1.5 software (OptiTrack, NaturalPoint Inc.) and data were collected at 100 Hz. The 6 markers were used to create a rigid-body object representative of each foot to track gait throughout the experiment. Participants wore a custom-designed safety harness at all times (same as originally presented in De Sanctis et al., 2014). Before experimental blocks, participants chose their preferred treadmill walking speed, and this was set for all subsequent walking experimental blocks. Three individuals in the ASD group do not have motion-tracking data and 2 had partial data. These were due to either software difficulties during setup, or poor motion-tracking recording quality and are therefore excluded from related analyses, creating group sizes of *n* = 17, or *n* = 16 for the W-F-T condition, in the ASD group and *n* = 18 in the TD group. For each participant, mean step width, mean stride length, mean stride time, and coefficients of variation (CV%) respective to those three measures were calculated.

### EEG recording, preprocessing, and analysis

Electroencephalography (EEG) was recorded from 64 channels with the BioSemi ActiveTwo system (BioSemi, Amsterdam, the Netherlands), following the international 10–20 system. The sampling frequency was 512 Hz. All EEG processing and analyses were performed using custom MATLAB scripts (MathWorks Inc., Natick, MA, United States) and functions from EEGLAB [99] or FieldTrip [100] toolboxes. Continuous EEG data were bandpass filtered at 0.25–40 Hz, applied using zero-phase filtering (“filtfilt” in EEGLAB) and hamming window types. The average reference was used. Independent component analysis was performed using the *runica* algorithm (“pop_runica“). Bad channels were automatically detected and manually confirmed, then interpolated using the spherical method. Trials were sorted based on type, Go or NoGo, and experimental condition, S-NF, W-NF, or W-F, based on triggers in the raw data. Epochs were time-locked to the onset of stimulus presentation and extended from 100 ms preceding stimulus onset (-100 ms) to 800 ms after, for a total epoch window of 900 ms.

### Individual factor measures

All individual factor measures were compared across groups and reported in Table 1.

- *Demographics*: Age, sex, handedness, race, and ethnicity were collected by self-report surveys for all participants. For children, a parent or guardian completed surveys on their behalf. Demographics are reported in Table 1.
- *Biometrics*: For all participants, height and weight were recorded. This was to ensure a normal distribution of body types in the groups.
- *Intelligence Quotient (IQ)*: IQ was determined with one of three Wechsler tests: Wechsler Intelligence Scale for Children-5th Edition (WISC-V), Wechsler Adult Intelligence Scale-4th Edition (WAIS-IV), or Wechsler Abbreviated Scale of Intelligence-2nd Edition (WASI-II). The WISC-V was used for those younger than 17 years old, while the WAIS-IV or WASI-II were used for individuals older than 17 years. Whether the WAIS-IV or WASI-II was used depended on which test (if either) had been administered to a participant during a previous HCP study visit within the past two years. If the participant did not have any Wechsler testing done with the HCP in the previous two years, the WASI-II was administered. Full-scale IQ (FSIQ) scores are able to be obtained from these 3 measures, and this score is used in the current study. Two individuals in the TD group do not have FSIQ scores.

All individual factor measures descriptives for both groups are reported in Table 2. Average age and non-verbal IQ scores did not differ between groups. The ASD group was heavily male-dominated relative to the control group, though both groups had more males than females.

**Table 1:** Group demographics

	TD		ASD	
	*N*	%	*n*	%
*SEX*				
Female	7	38.89%	3	15%
Male	11	61.11%	17	85%
*HANDEDNESS*				
Ambidextrous	1	5.56%	0	-
Left	1	5.56%	6	30%
Right	16	88.89%	14	70%
*RACE*				
Asian	1	5.56%	0	-
Black / African American	4	22.22%	8	40%
Multi-racial	3	16.67%	2	10%
Unknown/Prefer not to answer	0	-	3	15%
White	10	55.56%	7	35%
*ETHNICTY*				
Hispanic	5	27.78%	8	40%
Non-Hispanic	13	72.22%	12	60%

**Table 2:** Group descriptives

Measure	Group	*n*	Mean	Std. Deviation	Range	*p*
Age (years)	TD	18	17.19	2.58	13–23	0.610
	ASD	20	16.75	2.73	13–22	
Height (feet)	TD	18	5.64	0.32	5.10–6.20	0.755
	ASD	20	5.68	0.41	5.11–6.50	
Weight (lbs)	TD	18	146.33	24.49	100–183	**0.014**
	ASD	20	174.40	40.65	127–270	
Verbal IQ	TD	16	108.94	12.09	82–130	**0.014**
	ASD	19	95.79	17.74	72–136	
Non-Verbal IQ	TD	16	108.50	10.95	90–126	0.060
	ASD	19	99.47	15.57	81–140	

Ind. Samples t-tests. Bolded *p* values indicate significance at the 0.05 level.

### Statistical analyses

#### Cognitive task & behavioral performance

Cognitive task performance was measured by both accuracy and response time (RT). Accuracy was calculated using d’ (d’ = z-score (false alarm rate) – z-score (hit rate)). Response time is defined as the time between onset of stimulus presentation and the button press on successful Go trials (Hits). A mean d’ and RT was calculated for each participant during each of the 3 task-inclusive experimental conditions, S-NF-T, W-NF-T, and W-F-T. A repeated measures ANOVA in SPSS (IBM SPSS Statistics for Windows, Version 29.0.2.0 Armonk, NY: IBM Corp) was used to assess differences in d’ and RT on the vGNG task within (Condition: S-NF, W-NF, W-F) and between (Group: TD and ASD) groups. Notably, as there was no sensory-cognitive load experimental condition (no S-F-T), we could not perform repeated-measures ANOVAs with a 2 (Group) * 2 (Motor Load) * 2 (Sensory Load) design, which would be more ideal to control for multiple comparisons. Values reported are Greenhouse-Geisser corrected for violations of sphericity where appropriate. Effect sizes are partial eta squared. Post-hoc pairwise comparisons were conducted with Bonferroni’s correction to explore any significant effects and are reported with p-values, means and standard deviations, and Cohen’s *d*.

#### EEG

Custom MATLAB and EEGLAB scripts were used to process the data and extract dependent measures. Grand average ERPs and scalp topography plots were calculated by averaging all participants’ processed EEG data within each trial type and experimental condition. For topoplots, we calculated each group’s average peak latency for each ERP component. After calculating those, we centered across ± 25 ms for P2 and N2, and ± 50 ms for the P3, in two 50 ms segments, because the P3 is a more prolonged ERP. For ERP waveforms, electrode locations that were used to plot the ERPs were chosen a priori due to their well-characterized loci on the scalp and probable neural generators during the vGNG. These were FCz, Cz, and CPz (see scalp map in Fig. 1) [28, 86]. Difference ERPs were calculated by subtracting a group’s grand average for Hits from its grand average for CRs, respective to each of the 3 task-inclusive experimental conditions.

For statistical analyses, each participant’s average peak amplitude and latency were calculated for the P2, N2 and P3 within each of the 3 experimental conditions during both Hits and CRs. Each participant’s component peaks were calculated at the electrode location best reflecting its well-characterized scalp location, within respective latency windows well-characterized by prior literature, as discussed in the Introduction. For the P2, FCz was chosen and individual ERPs were found within the 200–280 ms window [79]. For the N2, we used FCz during the latency window within 280–380 ms [79, 80]. For the P3, CPz was used from 350 to 500 ms [4, 28, 79]. Chosen latency windows and channels were visually confirmed by study personnel using the group grand averages.

To statistically compare ERPs, repeated measures ANOVAs were performed at each component’s respective chosen electrode location with 2 (Response Type: Hits and CRs) * 3 (Condition: S-NF, W-NF, and W-F) within factors design, and between factor of Group (TD and ASD). For exploratory post-hoc analyses, cluster-based permutation tests were used [101]. 

We first compared Hits and CRs to determine the extent of inhibitory control’s impact on ERPs under each experimental condition and for each group. To do so, we did a 2*2 repeated measure ANOVA with factors of Condition with 3 levels: S-NF-T, W-NF-T, and W-F-T, and Response type with 2 levels: Hits or CRs, and between-subjects factor of Group. This was done separately for each component. Where main or interaction effects were found, the reported significance is after Greenhouse-Geisser correction if sphericity was violated, and effect sizes are reported as partial eta squared.

#### EEG exploratory analyses

To further explore the effects within this dataset, statistical t-value cluster-based permutation analyses were also used. Pointwise two-tailed paired t-tests were applied across each sample of the epoch window for each electrode. A cluster was considered significant only if consecutive points exceeded the set α = 0.05 threshold for a minimum of 10 consecutive sample points [102]. These tests allow for a more holistic perspective on the neurophysiology by providing a method to look at the full scope of the data across time and scalp location. Statistical cluster plots allow increased breadth with which to comprehensively characterize the effects observed while still controlling for multiple comparisons [4, 28, 30]. As an exploratory approach, the cluster analyses are interpreted cautiously and serve as complementary to the hypothesis-driven highly constrained a priori statistical analyses that only consider data from specific channels at specific time-windows, and to generate hypotheses for follow-up studies.

This approach has been previously used to analyze EEG data, including previous MoBI [28, 30, 103] and autism [104, 105] studies. The cluster analyses were done comparing the amplitude of the response between these two conditions for each task-inclusive experimental condition, S-NF-T, W-NF-T, and W-F-T, for both the TD group and the ASD group, creating a total of 6 cluster analyses.

A subsequent set of cluster analyses focused on group differences in the degree of impact on neurophysiology due to response inhibition during CRs. We aimed to analyze how motor and sensory loads impacted the neurophysiology of the two groups and investigate how response inhibition differed between groups. To do so, we calculated the difference of the responses between CRs and Hits within each group, and then compared the magnitude of the groups’ differences to each other within the cluster analyses, for each of the task inclusive experimental conditions, creating 3 cluster plots.

#### Motion-tracking and gait kinematics

Based on previous studies, the following quantitative gait markers were chosen which have been found to be sensitive measures to characterize gait stability in augmented or virtual reality environments: stride time, stride length, and step width [4, 28, 30, 98]. Heel strikes were computed from the heel (calcaneus) marker trajectory, using MATLAB custom scripts and the peak detection function, *findpeaks*. Identified peaks were confirmed by visual inspection, to ensure the identified peak was the point in the trajectory where the heel marker was at the most anterior position in the anterior-posterior direction (parallel to the direction of treadmill belt movement) [98]. Individual strides were defined as consecutive heel strikes of the same foot. Stride time was defined as the time between consecutive heel strikes of the same foot [106]. Step width was computed as the lateral distance (on the axis perpendicular to the direction of treadmill belt movement) between the two heel markers at the time of right heel strike [107]. Heel lift was used to define stride length in addition to heel strike, and was defined as the most posterior value on the anterior-posterior axis of the heel marker within an individual stride. Stride length was then defined as the distance between the location of a heel lift to the next heel strike, on the anterior-posterior axis. The means and coefficients of variance (CV% = (SD/mean) × 100) of each of these measures were calculated over each block of each condition, for every participant [30]. This created 6 dependent variables for gait for each participant. A 2 × 2 repeated measures ANOVA was completed respective to each dependent variable, with the factors of optical flow (No Flow or Flow), and cognitive task (No Task or Task), with an α = 0.05 and effect sizes reported with partial eta squared. Individual pairwise comparisons were used for *post-hoc* analyses, with Bonferroni correction to adjust for multiple comparisons, and effect sizes were estimated using Cohen’s *d*.

## Results

### Behavioral performance

**Fig. 2:**
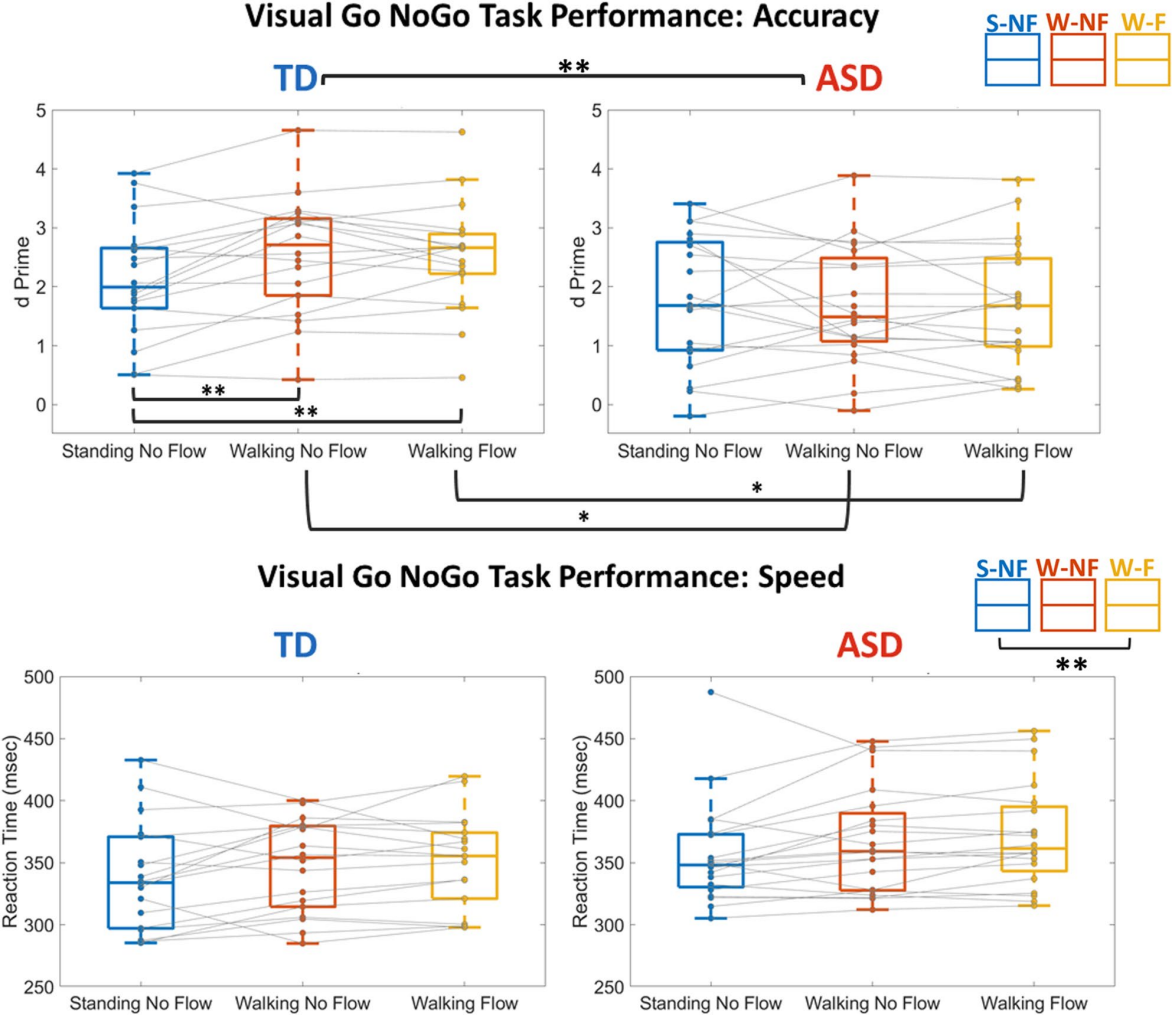
Within and between group differences in accuracy (d’) & reaction time (ms) on the visual Go-NoGo task. Each dot is one participant and a single participant’s performances across each experimental condition is connected by a gray line. Significant differences are marked by * = *p* < .05, ** = *p* < .01, *** = *p* < .001. On each box, the central mark is the median, and the edges of the box are the 25th and 75th percentiles. Any outliers are plotted individually outside their respective boxplot

Figure 2 illustrates participant performance on the vGNG task across experimental conditions. For reaction times, the repeated measures ANOVA revealed a main effect of Condition *F*(2,36) = 8.157, *p* = .003, η_p_^2^ = 0.185. *Post-hoc* pairwise comparisons with Bonferroni correction found that RT was significantly slower in the W-F (mean = 361.93 ± 40.95 ms) condition compared to the S-NF (mean = 348.02 ± 42.63 ms) condition (*p* = .005, d = 0.412), but the S-NF and W-NF comparison was not significantly different (*p* = .058, d = 0.241).

Analyses of task accuracy (d’) demonstrated a main effect of Group, *F*(2,36) = 4.944, *p* = .033, η_p_^2^ = 0.121. *Post-hoc* pairwise comparisons with Bonferroni correction found that the TD group (mean = 2.39 ± 0.98) was significantly more accurate than the ASD group (mean = 1.702 ± 0.95), (*p* = .033, d = 0.718). There was an interaction between Group and Condition, *F*(2,36) = 2.386, *p* = .012, η_p_^2^ = 0.124. *Post-hoc* pairwise comparisons with Bonferroni correction showed that the TD group (mean = 2.55 ± 1.01) was significantly more accurate than the ASD group (mean = 1.68 ± 1.00) in the W-NF (*p* = .012, d = 0.865) and the W-F (TD mean = 2.52 ± 0.94, ASD mean = 1.67 ± 0.1.04, *p* = .12, d = 0.858) conditions. Within groups, the ASD group showed no significant differences across conditions. The TD group had significantly higher accuracy for both the W-NF (mean = 2.55 ± 1.01) compared to S-NF (mean = 2.11 ± 0.987), (*p* =. 018, d = 0.438), and the W-F (mean = 2.52 ± 0.940) condition compared to S-NF (*p* = .017, d = 0.419).

### EEG & neurophysiology

#### Hit vs. CR responses in TD and ASD

**Fig. 3:**
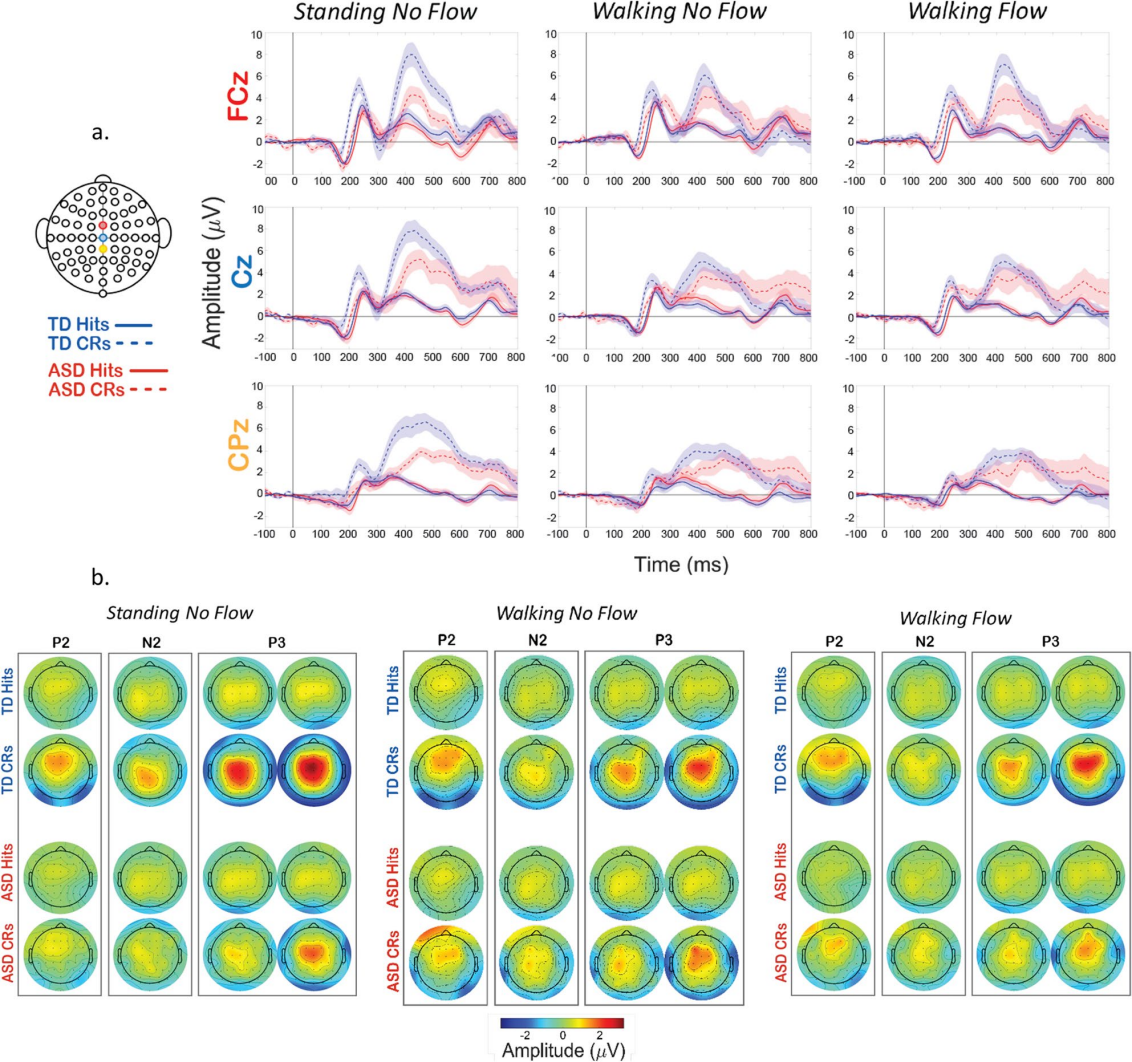
Grand average ERPs and topoplots for all conditions, trial types, and both groups across. **A** Grand average ERPs. Response type is represented by line style (Solid = Hits, Dashed = CRs) and group is represented by line color (Blue = TD, Red = ASD). Electrodes represented are FCz (top, red), Cz (middle, blue) or CPz (bottom, yellow). Font color of the electrode names matches color of the location of that electrode on the scalp map on the left-hand side. Shaded areas of the ERP waveforms are SEM. **B** Grand average topoplots. For the P2 and N2, the topoplot is centered ± 25 ms on that group’s average peak latency. For the P3, the first topoplot is -50 ms of the group’s average peak latency and the second is + 50 ms

**Fig. 4:**
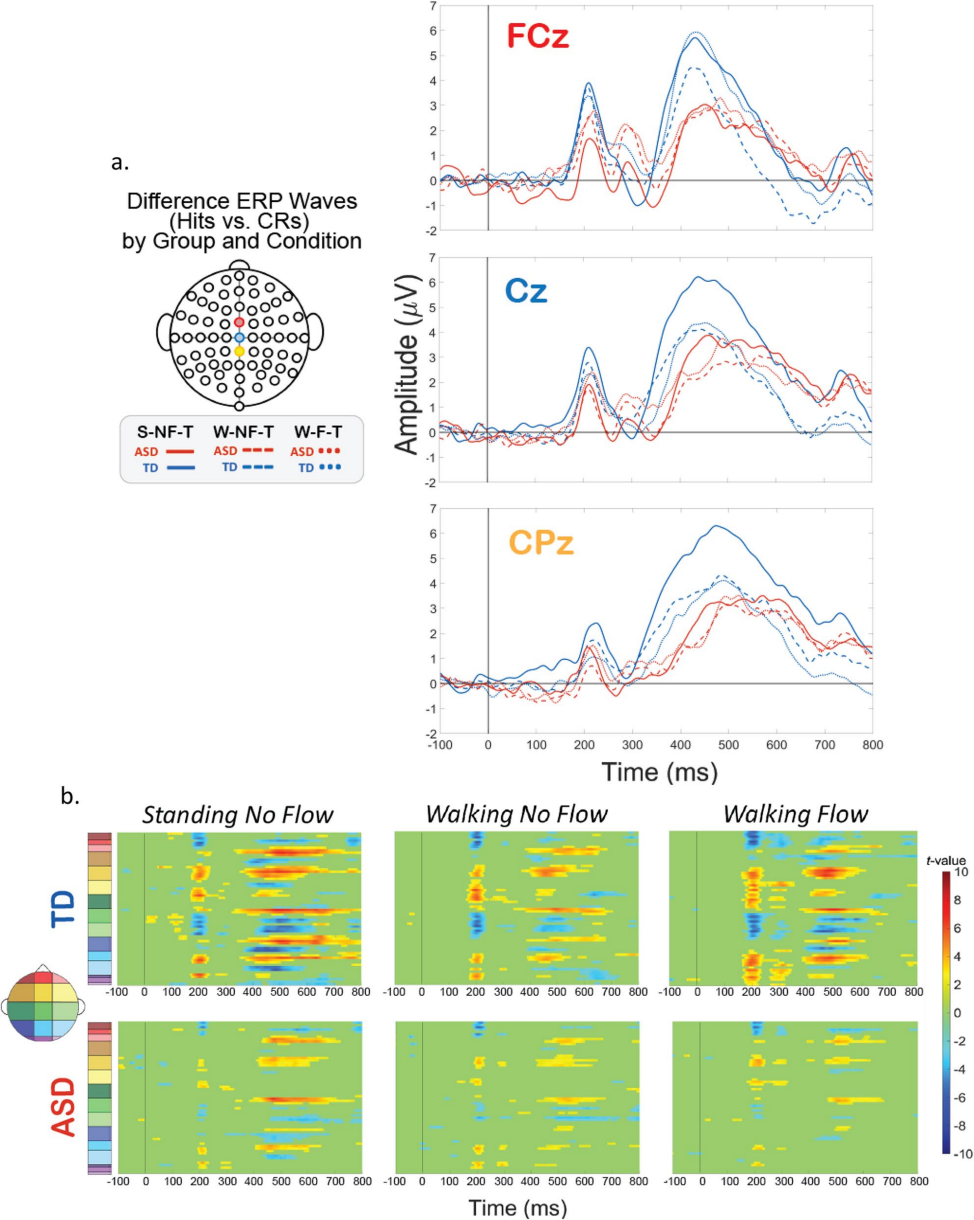
Experimental condition difference waves (Hits vs. CRs) for TD and ASD groups. **A**. ERPs difference waves calculated by taking the difference of ERPs for Hits (correct response during Go trials) and for Correct Rejections (“CRs”, correct withholding of a button press during NoGo trials). Blue lines represent difference ERPs for the TD group, and red lines represent participants with ASD. Line style represents experimental condition: solid for S-NF-T, dashed for W-NF-T, and dotted for W-F-T. Different plots indicate electrode: FCz (top, red), Cz (middle, blue) or CPz (bottom, yellow). **B**. Statistical cluster plots. A significant cluster indicates a statistically significant difference between Hits and CRs for that respective plot’s population and experimental condition. Y-axes colors correspond with scalp location as depicted by the colors on the scalp map on the left-hand side

Grand average ERP waveform (Fig. 3a) and scalp topography plots (Fig. 3b) show clear P2/N2/P3 responses were evoked in both groups for all conditions, and it can be seen that the magnitude of the evoked potentials differs as dual- (walking) and tri-modal (walking with optical flow) demands are introduced. Repeated measures ANOVAs for the peak amplitudes and latencies at predetermined electrodes and latency windows of interest were performed, as described in the methods. For the TD group, peak values were selected from 219 to 269 ms for the P2, 294–344 ms for the N2, and 363–463 ms for the P3. For the ASD group the windows were slightly adjusted to 225–275 ms for the P2, 296–346 ms for the N2, and 355–455 ms for the P3. Significant main and interaction effects are reported in Table 3 (peak amplitudes) and Table 4 (peak latencies). These significant effects are not marked on Fig. 3., to improve figure interpretability. For visual representation of the significant differences and as an exploratory approach to probing the full extent of the neurophysiological differences, we use statistical cluster plots in Fig. 4.

**Table 3:** *Peak Amplitudes* - Significant results of repeated measures 3 (Condition) * 2 (Response Type) ANOVAs on dependent variable *peak amplitude*. Greenhouse-Geisser corrected for violation of sphericity. Effect sizes are reported as partial Eta squared

Response Type	P2 Amplitude	N2 Amplitude	P3 Amplitude
	***F*(1**,**36) = 41.360**, ***p*** **< .001**,** η**_**p**_^**2**^ **= 0.535**	*F*(1,36) = 1.235, *p* = .274, η_p_^2^ = 0.033	***F*(1**,**36) = 65.204**, ***p*** **< .001**,** η**_**p**_^**2**^ **= 0.644**
Condition	*F*(1,36) = 2.405, *p* = .108, η_p_^2^ = 0.063	***F*(1**,**36) = 5.198**, ***p*** **= .014**,** η**_**p**_^**2**^ **= 0.126**	***F*(1**,**36) = 10.617**, ***p*** **< .001 η**_**p**_^**2**^ **= 0.228**
Group	*F*(1,36) = 3.073, *p* = .088, η_p_^2^ = 0.079	*F*(1,36) = 1.768, *p* = .192, η_p_^2^ = 0.047	*F*(1,36) = 3.533, *p* = .068, η_p_^2^ = 0.089
Group * Response Type	*F*(1,36) = 1.535, *p* = .223, η_p_^2^ = 0.041	*F*(1,36) = 1.540, *p* = .223, η_p_^2^ = 0.041	***F*(1**,**36) = 8.704**, ***p*** **= .006**,** η**_**p**_^**2**^ **= 0.195**
Group * Condition	*F*(1,36) = 0.981, *p* = .367, η_p_^2^ = 0.027	*F*(1,36) = 0.649, *p* = .426, η_p_^2^ = 0.018	*F*(1,36) = 1.515, *p* = .229, η_p_^2^ = 0.040
Response Type * Condition	*F*(1,36) = 2.989, *p* = .316, η_p_^2^ = 0.032	***F*(1**,**36) = 3.475**, ***p*** **= .039**,** η**_**p**_^**2**^ **= 0.088**	*F*(1,36) = 2.540, *p* = .086, η_p_^2^ = 0.066
Group * Response Type * Condition	*F*(1,36) = 1.005, *p* = .371, η_p_^2^ = 0.027	*F*(1,36) = 0.124, *p* = .874, η_p_^2^ = 0.003	*F*(1,36) = 0.802, *p* = .452, η_p_^2^ = 0.022

**Table 4:** *Peak Latencies* - Significant results of repeated measures 3 (Condition) * 2 (Response Type) ANOVAs on dependent variable *peak latency*. Greenhouse-Geisser corrected for violation of sphericity. Effect sizes are reported as partial Eta squared

Response Type	P2 Latency	N2 Latency	P3 Latency
	***F*(1**,**36) = 8.463**, ***p*** **= .006**,** η**_**p**_^**2**^ **= 0.190**	***F*(1**,**36) = 15.138**, ***p*** **< .001**,** η**_**p**_^**2**^ **= 0.296**	***F*(1**,**36) = 50.815**, ***p*** **< .001**,** η**_**p**_^**2**^ **= 0.585**
Condition	*F*(1,36) = 0.386, *p* = .681, η_p_^2^ = 0.011	*F*(1,36) = 1.506, *p* = .229, η_p_^2^ = 0.040	***F*(1**,**36) = 3.302**, ***p*** **= .043**,** η**_**p**_^**2**^ **= 0.084**
Group	*F*(1,36) = 1.662, *p* < = 0.206, η_p_^2^ = 0.044	*F*(1,36) = 0.116, *p* = .736, η_p_^2^ = 0.003	*F*(1,36) = 0.289, *p* = .594, η_p_^2^ = 0.008
Group * Response Type	*F*(1,36) = 0.217, *p* = .644, η_p_^2^ = 0.006	*F*(1,36) = 4.064, *p* = .051, η_p_^2^ = 0.101	*F*(1,36) = 0.441, *p* = .551, η_p_^2^ = 0.012
Group * Condition	***F*(1**,**36) = 3.831**, ***p*** **= .027**,** η**_**p**_^**2**^ **= 0.096**	*F*(1,36) = 1.625, *p* = .205, η_p_^2^ = 0.043	*F*(1,36) = 0.269, *p* = .764, η_p_^2^ = 0.007
Response Type * Condition	*F*(1,36) = 1.407, *p* = .251, η_p_^2^ = 0.038	*F*(1,36) = 0.792, *p* = .451, η_p_^2^ = 0.022	*F*(1,36) = 0.897, *p* = .406, η_p_^2^ = 0.024
Group * Response Type * Condition	*F*(1,36) = 3.047, *p* = .055, η_p_^2^ = 0.078	*F*(1,36) = 0.164, *p* = .837, η_p_^2^ = 0.005	*F*(1,36) = 0.020, *p* = .975, η_p_^2^ = 0.001

With consideration for readability, the pairwise post-hoc comparisons are not included in the tables, but are delineated below. For the P2, CR amplitudes were significantly stronger than Hits (*p* < .001), and CR latencies were significantly shorter than hits (*p* = .006). The Group * Condition interaction for the P2 latency did not maintain significance in any *post-hoc* pairwise comparisons. At FCz, the N2 amplitude was significantly reduced in the W-NF condition compared to the S-NF condition (*p* = .030). Within the W-F condition, CRs were significantly greater than hits (*p* = .044). The N2 CR latency was significantly longer (*p* < .001) than that of Hits. For the P3, CR amplitudes were significantly stronger (*p* < .001), with longer latencies (*p* < .001) than Hits. For the main effect of condition, S-NF condition amplitude was significantly stronger than the W-NF (*p* = .005) and the W-F (*p* = .002) conditions. For the interaction of Group * Response Type, CRs were significantly stronger in amplitude for the TD group (*p* = .017). The latency main effect of Condition did not hold significance *post-hoc*.

### Exploratory analyses

To further statistically explore the relationships amongst these factors, we next analyzed the magnitude of the difference between Hits and CRs for each group and condition (Fig. 4). We performed t-statistic cluster-based permutation analyses, which allows for a comparison of all points on the scalp across all times in an epoch. A cluster is formed only by 10 or more consecutive significantly different time points between Hits and CRs. This approach resulted in clusters of statistical difference during the P2 latency window and the P3 latency window (Fig. 4). Clusters are seen from 200 to 280 ms over frontal-central regions (yellow and green regions on the scalp map), in each group and condition, though to a considerably stronger extent for the TD group. Similarly for the P3, clusters emerge at approx. 350 ms over central-parietal (green, and blue) and also more fronto-central areas (yellow) for each group and condition, but to a much stronger extent in the TD group. Clusters during the N2 window, approx. 280–380 ms, are minimal, except in the TD’s W-F-T condition, where centrally-located clusters emerge, indicative that this stage of processing was impacted by the tri-modal condition.

When comparing the group differences in intensity of these clusters in terms of number of channels and durations of significant time points, an interesting pattern emerged. For the ASD group, the clusters lessened with increased load, wherein the strongest difference (highest durations and numbers of statistical clusters) occurred during S-NF-T blocks (single modal), was weaker in the W-NF-T blocks (cognitive-motor dual modal), and was weakest in the W-F-T (motor-sensory-cognitive tri-modal). However, for the TD group, the pattern was not incremental like the ASD group. The strongest effect was still during the S-NF-T blocks, and was weakened in the W-NF-T blocks, but to a lesser extent in the W-F-T blocks. In every condition, the TD group showed stronger differences between Hits and CRs than the ASD group.

#### Multi-Modal processing and inhibitory control: TD vs. ASD

**Fig. 5:**
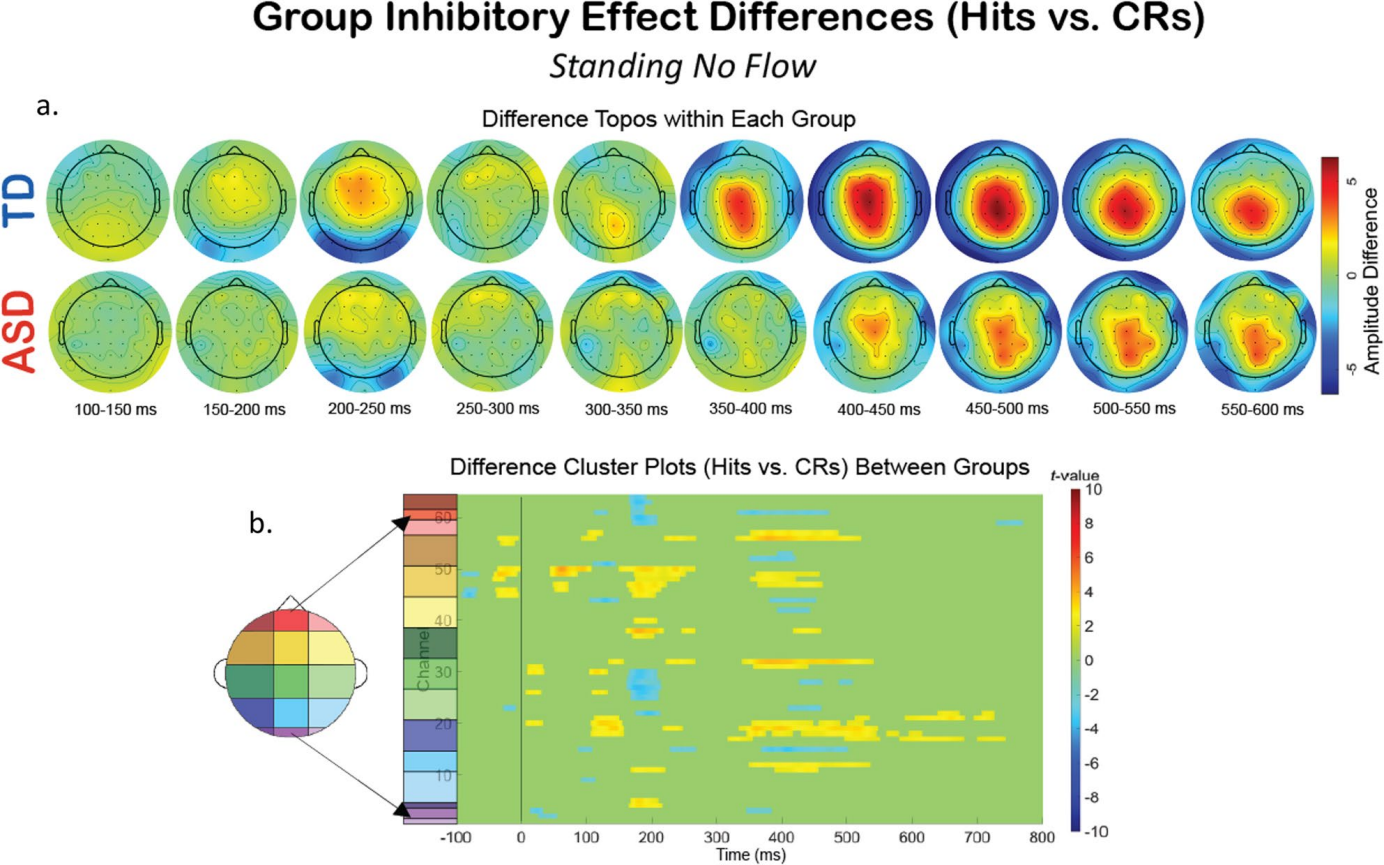
The difference in EEG during the cognitive-only experimental condition, S-NF-T, across response types (Hits vs. CRs). **A** Difference topoplots where each plot is the difference in activity between Hits and CRs for that group and time segment. **B** Cluster-based analysis plots compare differences between groups’ NoGo Effect (the difference between Hits and CRs

**Fig. 6:**
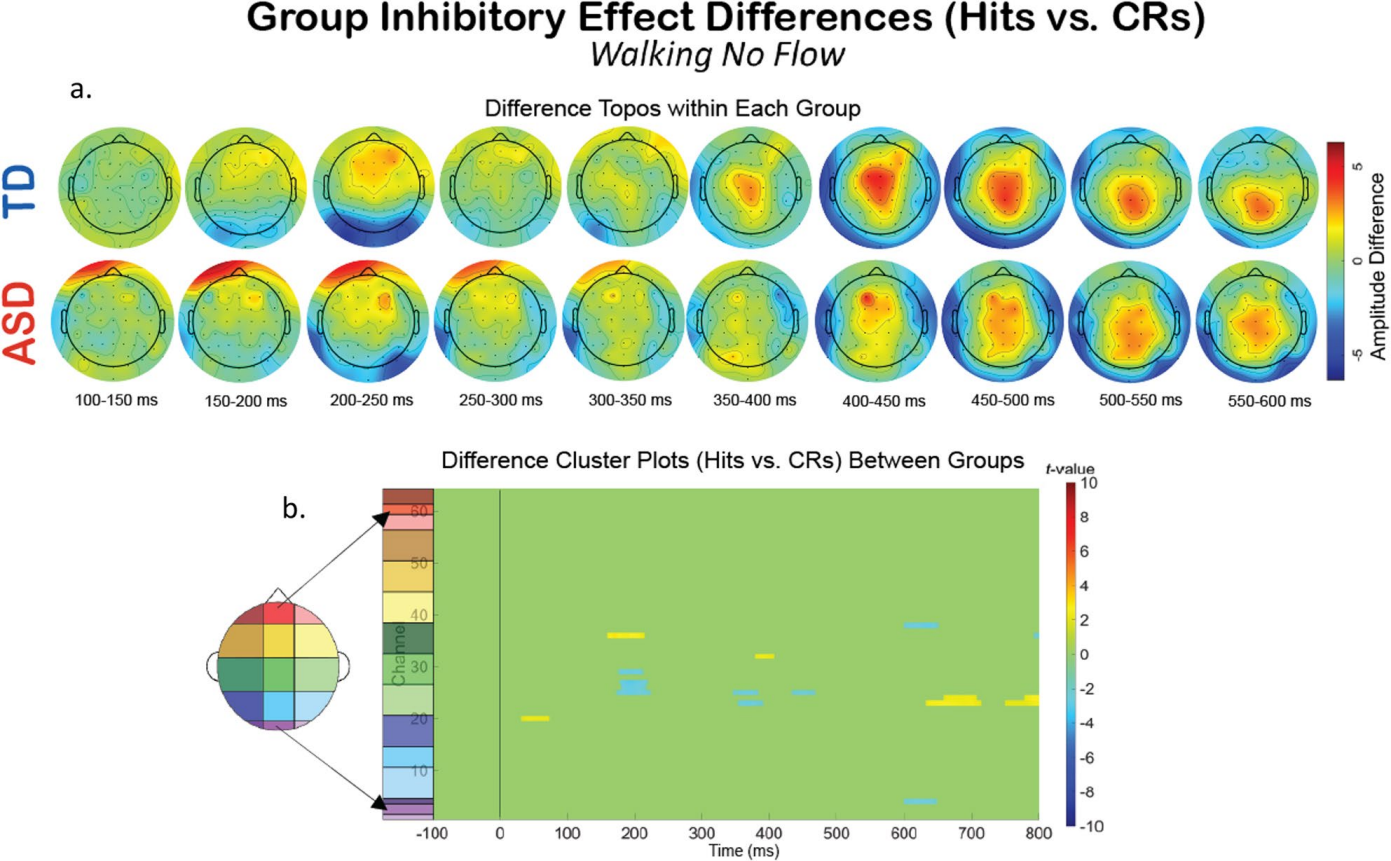
The difference in EEG during the cognitive-motor dual-modal experimental condition, W-NF-T, across response types (Hits vs. CRs). (**A**) Difference topoplots where each plot is the difference in activity between Hits and CRs for that group and time segment. (**B**) Cluster-based analysis plots compare differences between groups’ NoGo Effect (the difference between Hits and CRs)

**Fig. 7:**
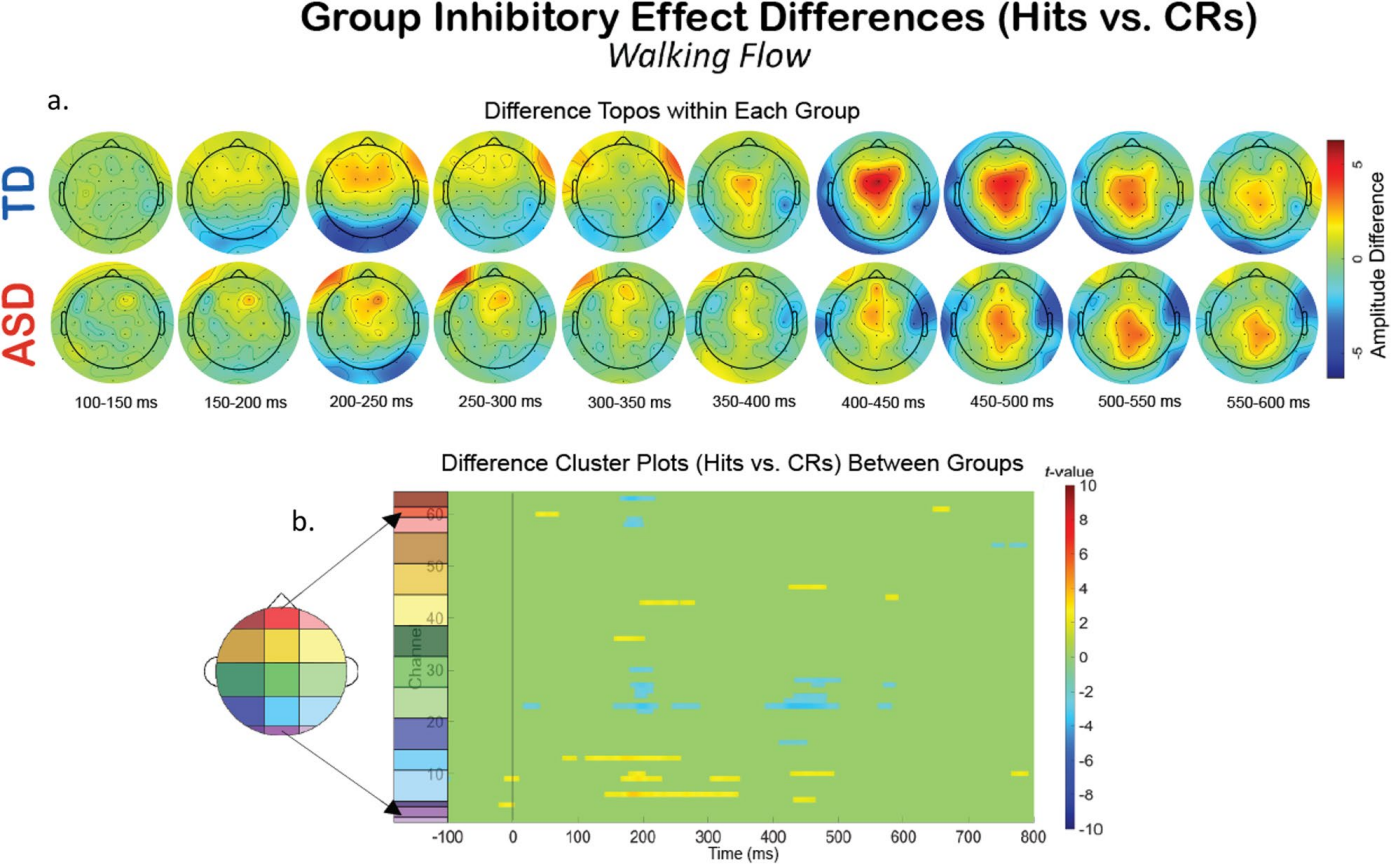
The difference in EEG during the motor-cognitive-sensory tri-modal condition, W-F-T, experimental condition across response types (Hits vs. CRs). (**A**) Difference topoplots where each plot is the difference in activity between Hits and CRs for that group and time segment. (**B**) Cluster-based analysis plots compare differences between groups’ inhibitory effect

Additionally, we compared the groups within a specific experimental condition, and examined how the extent of the impact of dual- and tri-modal demands on neurophysiology fluctuates differentially between the two groups. To explore this, we created difference topoplots. Difference topoplots (Figs. 5a and 6a, and 7a) subtracted the grand average scalp topography for Hits from the grand average for CRs within each group in 50 ms segments from 100 ms to 600 ms. Positive amplitude differences (warm colors) on the difference topoplots are indicative of greater amplitudes during CRs. We then used the same statistical cluster-plot approach as in Fig. 3., but now compared the difference in the extent of each groups’ difference between response types. The cluster plots show the statistical difference between the magnitude of the change across response types (CRs minus Hits) in the TD group compared to the magnitude of the same change in the ASD group within each of the S-NF-T (Fig. 5.), the W-NF-T (Fig. 6.), and the W-F-T (Fig. 7.) conditions.

S-NF-T: During the cognitive-only condition (Fig. 5.), both the difference topoplots and the statistical cluster plots show greater inhibitory effects in the TD group in the time windows and scalp locations corresponding to the ERP component of interest, the P2 (approx. 200–250 ms over fronto-central areas) and P3 (approx. 350–550 ms over central-parietal scalp regions). The P2 stage reflects attentional processing, and at the P3 stage the motor and cognitive planning components of inhibition are executed. Significant clusters here suggest that the TD group has a stronger difference between CRs and Hits during these stages than the ASD group. The difference between CRs and Hits for the N2 appears similar in magnitude for both groups, evidenced by minimal significant clusters in that time window (approx. 250–350 ms) over fronto-central (mid-yellow and mid-green on the scalp map) regions). The lack of difference clusters here suggests that at the conflict monitoring stage of processing, the groups are similar in monitoring inhibitory conflict when standing and without flow.

W-NF-T: During the cognitive-motor dual-modal condition, the difference topoplots show stronger differences between hit and CRs for the TD group than the ASD group during the P3 time window. However, the statistical cluster plots show very few significant clusters, indicating that the extent of inhibitory impact is very similar between the groups when walking and doing the task, but without any optical flow. (Fig. 6)

W-F-T: When optical flow is introduced, creating the sensory-cognitive-motor tri-modal condition (the highest load condition), the TD and ASD group showed slightly more significant clusters than in the W-NF-T condition, but not to nearly the same extent as they did in the S-NF-T condition. (Fig. 7)

### Gait kinematics

Chosen walking speed on the treadmill was not recorded for every participant, but for those it was recorded, the average walking speed for the TD group (*n* = 12) was 1.89 (± 0.329) miles per hour (0.845 m per second), and for the ASD group (*n* = 11) the average was 1.47 (± 0.531) miles per hour (0.657 m per second). These speeds were significantly different from each other, (t_21_ = -2.296, *p* = .032, Cohen’s *d* = 0.959), with the ASD group choosing a slower walking speed. Walking speed was not significantly correlated with age (Pearsons *r* = .324, *p* = .132), weight (Pearsons *r* = -.209, *p* = .339), nor height (Pearsons *r* = .368, *p* = .084). A 2 (Flow or No Flow) * 2 (Task or No Task) repeated measures ANOVA was performed for each of the six gait kinematic dependent variables. Where significant interactions or main effects are found, *F* statistics, *p* values, and effect sizes as partial eta-squared are reported. *Post-hoc* pairwise comparisons with Bonferroni correction are reported, with effect sizes as Cohen’s *d*.

For Step Width, there was a main effect of flow, *F*(1,33) = 42.258, *p* < .001, η_p_^2^ = 0.569. *Post-hoc* comparisons showed that step width was significantly wider (*p*_bonf_ < 0.001, d = -0.09) when there was optical flow (mean = 200.06 ± 37.63), compared to when there wasn’t flow (mean = 196.42 ± 37.41 mm). There was also a main effect of group, *F*(1,33) = 10.618, *p* = .003, η_p_^2^ = 0.249. *Post-hoc* pairwise with Bonferroni correction showed that step width was significantly wider (*p*_bonf_ =0.003, d = -1.10 mm) for those in the ASD group (mean = 218.89 ± 36.88) than those in the TD group (mean = 177.59 ± 38.01 mm).

Stride Time resulted in a main effect of flow, *F*(1,33) = 5.734, *p* = .022, η_p_^2^ = 0.148. *Post-hoc* comparisons showed that stride time was significantly (*p*_bonf_ =0.022, d = 0.055) shorter when there was optical flow (mean = 1298.25 ± 144.06 ms) compared to when there was no flow (mean = 1306.19 ± 146.26 ms).

For Stride Length, a main effect of flow was found, *F*(1,33) = 4.614, *p* = .039, η_p_^2^ = 0.126. *Post-hoc* pairwise with Bonferroni correction showed that stride length was significantly (*p*_bonf_ =0.039, d = 0.03) shorter when there was optical flow mean = 1230.15 ± 217.34 mm) compared to when there was no flow (mean = 1237.41 ± 223.26 mm). There was also a main effect of task, *F*(1,33) = 7.200, *p* = .011, η_p_^2^ = 0.184. *Post-hoc* comparisons showed that stride length was significantly (*p*_bonf_ =0.011, d = 0.07) shorter when participants were performing the task (mean = 1226.02 ± 230.19) compared to when they were not (mean = 1241.55 ± 210.90 mm). Further, there was an interaction of flow * task, *F*(1,33) = 6.472, *p* = .016, η_p_^2^ = 0.168. Stride length was the shortest in the F-T condition (mean = 1218.3 mm) compared to NF-NT (mean = 1241.1 mm), F-NT (mean = 1241.995 mm), and NF-T (mean = 1233.7 mm).

For CV% Step Width, we found a main effect of flow, *F*(1,33) = 6.113, *p* = .019, η_p_^2^ = 0.160.) *Post-hoc* pairwise with Bonferroni correction showed that step width was significantly (*p*_bonf_ =0.019, d = 0.137) less variable when there was optical flow (mean = 8.799 ± 3.011) compared to when there was no flow (mean = 9.231 ± 3.278). Additionally, there was a main effect of task, *F*(1,33) = 54.091, *p* < .001, η_p_^2^ = 0.628. *Post-hoc* comparisons showed that step width was significantly (*p*_bonf_ < 0.001, d = 0.135) less variable when participants were doing the task (mean = 8.395 ± 2.99) compared to when they were not (mean = 9.636 ± 3.29).

For CV% Stride Time, there was a main effect of task, *F*(1,33) = 28.149, *p* < .001, η_p_^2^ = 0.460. *Post-hoc* pairwise with Bonferroni correction showed that stride time was significantly (*p*_bonf_ < 0.001, d = 0.485) less variable when doing the cognitive task (mean = 2.860 ± 1.26) compared to when they were not (mean = 3.569 ± 1.64). A main effect of group was also found, *F*(1,33) 6.674, *p* = .014, η_p_^2^ = 0.168. *Post-hoc* comparisons showed that stride time was significantly (*p*_bonf_ =0.014, d = -0.873) more variable in the ASD group (mean = 3.830 ± 1.41) than the TD group (mean = 2.599 ± 1.41).

Lastly, for CV% Stride Length a main effect of task was found, *F*(1,33) = 16.693, *p* < .001, η_p_^2^ = 0.343. *Post-hoc* comparisons showed that stride length was significantly (*p*_bonf_ =0.014, d = 0.359) more variable when participants were not doing the task (mean = 3.074 ± 1.63) compared to when they were (mean = 2.554 ± 1.24). Additionally, we found a main effect of group, *F*(1,33) = 6.124, *p* = .019, η_p_^2^ = 0.161. *Post-hoc* comparisons showed that stride length was significantly more (*p*_bonf_ =0.019, d = -0.84) variable in the ASD group (mean = 3.400 ± 1.42) than the TD group (mean = 2.229 ± 1.38).

**Fig. 8:**
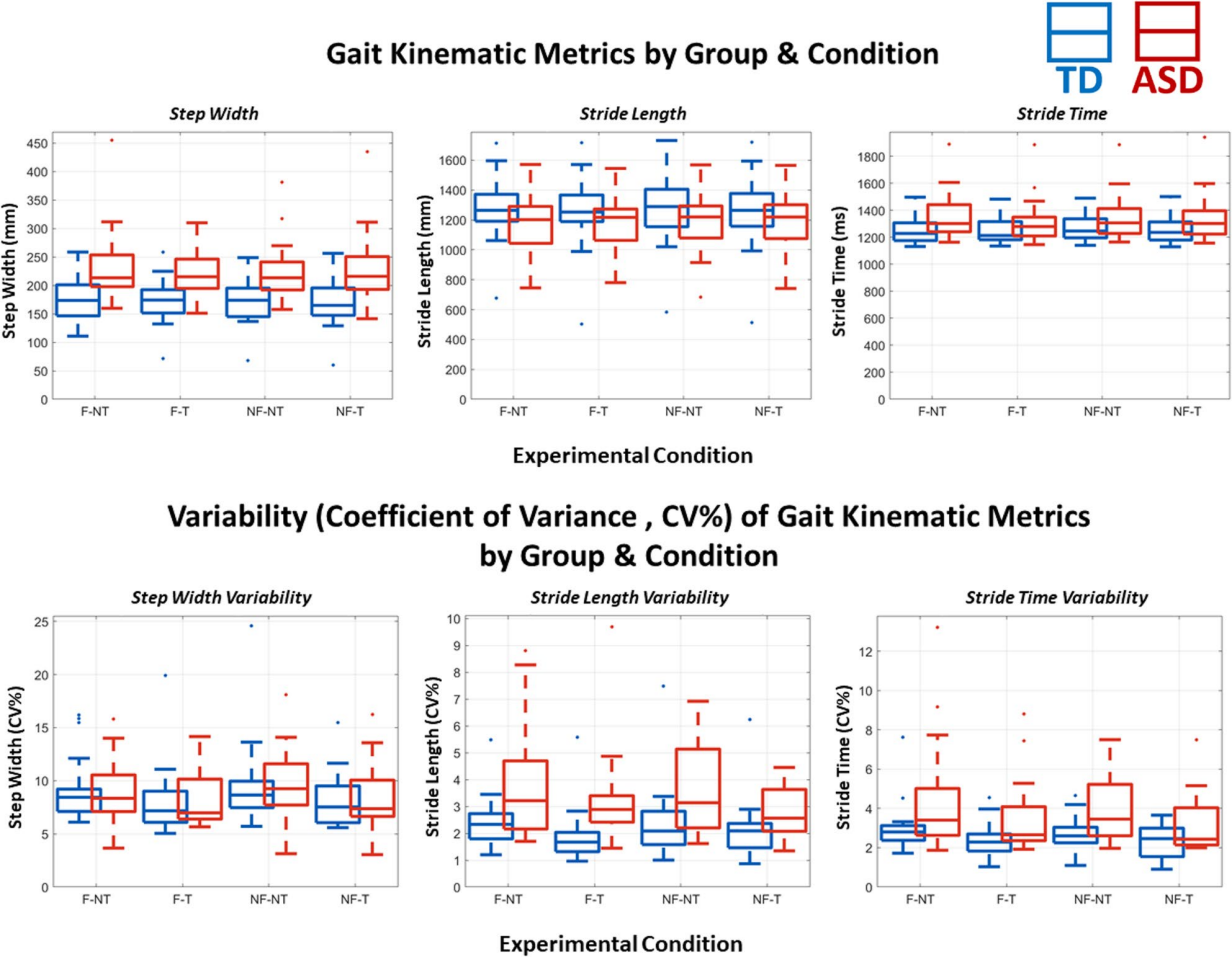
TOP ROW: Each of the 3 plots presents one of the gait kinematic metrics recorded: Step Width (left), Stride Length (middle) and Stride Time (Right). Within each plot are 8 boxplots. There is a TD/ASD boxplot pair for each condition to compare between groups. Within a plot, the conditions are ordered, from left to right, F-NT (Flow No Task), F-T (Flow Task), NF-NT (No Flow No Task), and NF-T (No Flow Task). Outliers are marked for each boxplot with a dot in the respective group’s color. BOTTOM ROW: The same as the top, except the three plots represent the coefficient of variance (CV%) respective to each gait kinematic metric

**Fig. 9:**
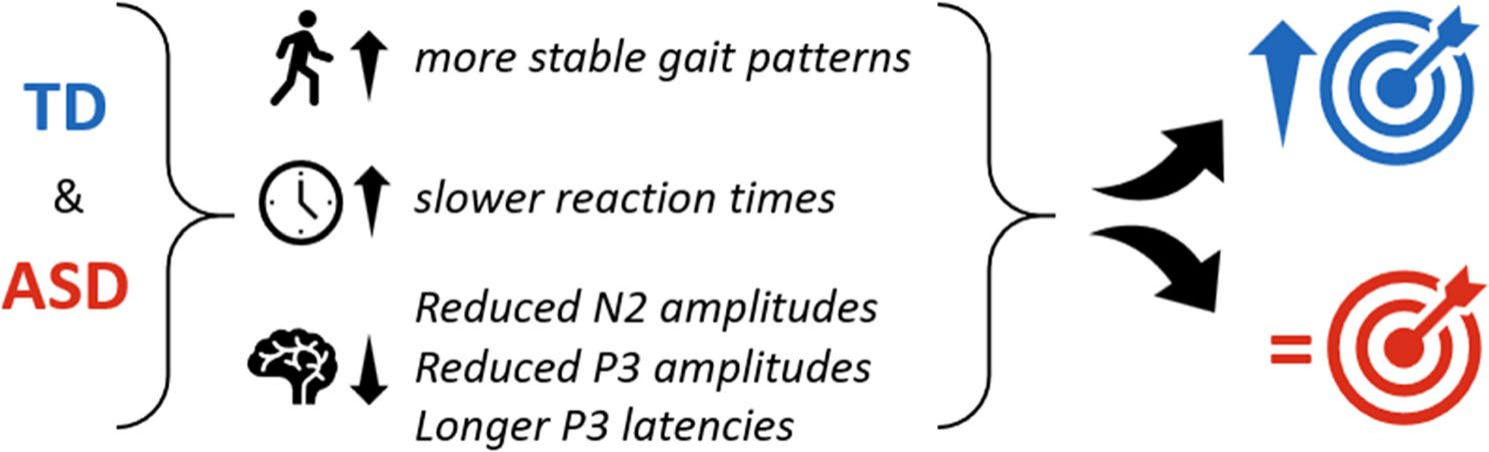
General, simplified results of the impact of dual- and tri-modal demands on gait, task performance, and neurophysiology in the TD and ASD groups

Table 5 provides a summary of all significant results (Figs. 8 and 9).

## Discussion

MoBI was used to assess changes in ERP characteristics under single-, dual, and tri–modal conditions, and compare the extent of flexibility of neurophysiological processes across populations in the context of a natural brain-body relationship, walking while performing a cognitive task and under changing sensory conditions. There is a significant gap in understanding these multi-modal interactions in younger ages. Investigating this in ASD is of particular import because this method promises to improve our ability to understand the underlying, real-time, neural mechanistic changes during dual- and tri-modal functioning, which is how we as humans most often function. To do so, we measured ERPs, gait kinematics, and cognitive task performance in adolescents with ASD and their typically developing counterparts. The current study employs MoBI in a novel age range (including 13–18 years old) and population (ASD), with the goal of characterizing the dynamic interplay between sensory, cognitive, and motor processing and function in adolescents.

### Group differences in response Inhibition task performance under different dual- and tri-modal conditions

We observed that the TD group was, overall, better at correctly withholding responses than the ASD group. As mentioned in the Introduction, there are known differences in inhibitory control between these groups. The literature is mixed, though, on the details. Some studies show deficits in ASD inhibitory control [35, 39, 108, 109], while others show no difference [89, 90], possibly reflecting specifics of the study design, such as which specific response inhibition task was used, whether co-morbid ADHD was considered, format of task presentation, and/or if the task included distractors [37, 109–112]. 

On our cognitive task, walking (both with and without optical flow) improved task accuracy for the TD group, but this was not the case for the ASD group. For typically developing individuals, this finding expands on similar ones in young adults showing a performance benefit when walking [28], and supports further study into the real-time benefits of walking in even younger ages. No studies, to our knowledge, have used EEG to characterize the underlying neural activity of this in those under 18 years old.

These results, in combination with the reaction time results showing increased speeds when walking, suggests that the TD group may have adopted a classic speed-accuracy tradeoff strategy [113] when walking with flow. However, when walking without flow, the increase in accuracy was maintained but the slowed reaction time was not implemented yet. It is possible greater variability in reaction time drove this, or that the TD group hadnt needed to adopt the strategy employed for the W-F-T condition that appears more similar to a speed-accuracy tradeoff until flow was introduced. Future studies could investigate relationships between reaction time and accuracy at the individual level to look for types of strategy adjustments and whether this differs as a function of age and/or group.

Of note is the lack of any significant accuracy changes in the ASD group across the experimental conditions, which is not necessarily suggestive of an impaired ability to adjust behavioral strategy under dual- and tri–modal conditions. These conditions, contrary to our expectations, did not decrease the accuracy of the ASD group either; they demonstrated maintenance. While it is possible a floor effect in ASD group during the S-NF-T condition could explain this, this is unlikely as their accuracies reflect good understanding of the task, well above chance (d’ = 0), and that is not different from their TD counterparts in that condition. This real-time accuracy maintenance for the ASD group could reflect a preference for not adopting alternative strategies and reallocating neural resources under dual- and tri–modal demands in a way that maintains their baseline approach, favoring consistency even under added demands across functional domains, which may also by extension explain the weaker EEG differences between motion states in the ASD group. There also could be greater variability in strategy in the ASD group than the TD group, especially with this study’s relatively small group sizes. There is a myriad of literature supporting cognitive flexibility and resource allocation impairments in ASD [69, 114], which helps contextualize our results, but the central mechanism is debated (as is the exact definition for “cognitive flexibility” and “resource allocation”). Some suggest that the rigidity and repetitive behaviors characteristic of ASD may underly this impairment, or vice versa [75, 76, 115]. Others point to processing speed [116] or lower cognitive control capacity [117, 118] as the foundation of difficulty with cognitive flexibility. Further, typically developing individuals may derive more of an arousing benefit from the physical activity of walking on concentration on our (admittedly tedious) cognitive task. There is likely no cardinal reason for cognitive flexibility differences, as none of these facets are mutually exclusive. The enduring question is whether the TD group’s improved performance suggests a higher threshold for multi-modal processing, or propensity for changing between different strategies in which resources are able to be flexibly redirected away from other processes, whereas the ASD group may be more likely to trend toward adaptations that result in consistent accuracy.

It should also be noted that at the individual level, there were improvers and worsen-ers in both groups. In line with the Patelaki et al. study, [28] this can possibly be attributed to individual ability to flexibly adapt to dual- or tri- modal demands. Alternatively, it has been shown that participant performance on similar cognitive control tasks can be influence by perceived challenge and motivation or incentive [119]. If the TD group did shift their strategy while the ASD group preferred maintenance, the TD group’s increase in performance could reflect greater effort investment and consequent greater perceived reward for more accurate performance.

Optical flow had no statistically significant impact on accuracy of the vGNG task for these participants. We did observe that walking (W-F-T) significantly increased reaction time for all participants, compared to standing (S-NF-T) but no significant adjustment to task speed was made prior to the addition of optical flow (W-NF-T). One possibility is that these adolescents were focusing their attention on the cognitive task, at a potential cost to monitoring of their peripheral environment. Especially because this optical flow was consistent and aligned with the direction of treadmill movement, it did not offer visual environmental information that was conflicting or perturbating, nor useful to maintaining performance of the other two tasks (walking and the vGNG). There were also no explicit instructions to attend to or to ignore the flow. It is possible the flow was easy for participants to disregard, and therefore it did not add a substantial enough load in the sensory domain to impact accuracy. One study has shown that performing a vGNG task while treadmill walking actually reduced the impact of perturbations in visual flow [98], further supporting the notion that there may be a lack of processing of the flow when attention is dedicated to the task. It is worth noting that there are reports showing higher susceptibility to distractor inputs in Autism [110, 120], whereas the current results suggest that the optical flow inputs used herein were not sufficiently distracting to tax this particular aspect of neural processing in either cohort. The omission of more challenging sensory stimuli, such as those used in Malcolm et al., 2018, was chosen because the MoBI recording experiment is already long and demanding on participants. The Malcolm study was conducted in healthy young adults, and did not include standing conditions. Since our study was exploratory in nature, it was critical to include the standing condition to establish a baseline comparison condition. Furthermore, we considered that congruent visual flow might be more taxing for ASD.

### Group differences in gait under different demand conditions

While not affecting task performance, optical flow did impact some of the gait metrics. Optic flow increased step width, and decreased stride time and length, and variance of step width. That is, all participants took wider steps and more consistent strides when there was optical flow included. Stabilizing gait through wider gait width has been reported in both TD [4, 13, 30] and ASD [44] groups, and is a way to feel more balanced by creating a wider base of support [44, 121], and is considered to reflect adapting a more cautious walking style [122]. These adjustments were made regardless of group in the current study, despite group differences on 3 of 4 of these impacted metrics as a whole (regardless of flow or task). The ASD group had wider steps and more variable stride lengths and times than the TD group. This is in line with previous literature, supporting that individuals with ASD have less stable and ‘smooth’ gait patterns [47]. We expanded on this with the current results and showed that, despite this, those with ASD make similar gait adaptations under multi-modal demand conditions. Possibly, the motor adaptations needed for stabilizing gait in this way in response to the introduction of optical flow prevented further neural flexibility to allocate resources to cognitive performance, explaining the lack of behavioral differences between the W-NF-T and W-F-T conditions in either group. An alternative point comes from a 2014 study from De Sanctis et al., who demonstrated a significant increase in stride time, but not stride time variability, when comparing two levels of walking speed [4]. Therefore, it is possible our main effect of condition on stride time is actually driven by the group difference in chosen walking speed on the treadmill. Though, walking speed was not recorded for all of our participants, and therefore was not used as a covariate as it would limit statistical power in this already small sample size. Further research should pursue these alternative explanations for gait adjustments under multi-modal demands in ASD using large sample sizes and perhaps multiple walking speeds (or other ways of dialing motor demand).

Engaging in the cognitive task decreased variability (CV%) of all gait metrics: stride time, stride length, and step width. Additionally, stride length was shorter when doing the vGNG task, compared to not. Similar results have been reported by other MoBI studies from our group in stride-to-stride trajectory variability [13], stride length and time variability [30] for cohorts of young adult participants. Stride time variability has also been shown in 8–13 year old children to decrease costs in a dual-task paradigm, though with considerable individual variability [116]. We replicate that here in a younger cohort and expand, providing new evidence across multiple gait kinematic metrics that cognitive-motor interactions similarly impact gait in adolescents, regardless of ASD diagnosis. This reduction in variability across metrics in previous studies implied a shift to a more automatic state of walking control [28, 123, 124], as is typical in everyday functioning [125], so more resources can be afforded to an additional task (in this case, the cognitive vGNG task). However, as discussed in the previous paragraphs, the ASD group neither benefited nor suffered behaviorally during walking. It is possible these gait adjustments, though not different from the TD group, did not afford the ASD group any reallocateable neural resources that could be directed toward the vGNG task. Or, alternatively, the reallocated resources in the ASD group were directed to maintaining task strategy instead of changing. One possible explanation for this is that the underlying mechanism of shifting to a more stable gait when engaging in the task may be indicative of a more typical, automated walking state for TD individuals, but is a more effortful shift for the ASD group. It has been demonstrated that increasing motor demands interfere with processing speed in children with ASD, but not their typically developing counterparts [74], which provides further evidence that there is a disrupted ability in ASD to adapt under dual- and tri-modal demands and that their capacity limits for multi-modal functions are more easily reached. However, we did not observe interference, per se, but rather a lack of the walking-based benefit that the TD group demonstrated. Therefore, again, the ASD and TD groups may simply have different tendencies in how their multi-modal adaptations are directed.

### Group differences in neurophysiology under different dual- and tri-modal conditions

We explored these possible interpretations further by looking at the neurophysiology related to walking in each group. Some brain areas heavily involved in walking are subcortical and therefore not as robustly characterized by EEG, like the cerebellum and basal ganglia [126]. One review of studies using functional near-infrared spectroscopy (fNIRS) suggests that as the degree of automaticity of motor control decreases (becomes less automatic), the involved cortical areas are more frontal-prefrontal, involving the premotor and supplementary motor areas, and the prefrontal cortex. A more automatic state would be more frontocentral, reflecting the primary motor cortex [127]. This adjustment would be evident in the EEG recordings as attenuations in the component peaks, as available neural resources are re-allocated to accommodate the addition of walking [4, 28, 30]. 

We observed that experimental conditions did impact the N2 and P3 peaks, the ERPs classically investigated with the GNG task, but only through the motor domain (walking), not the sensory domain (optical flow). For the N2, commonly associated with conflict monitoring in this task, the amplitude was reduced in the W-NF condition, suggesting the dual-modal condition was significantly demanding on the brain’s resources, but the addition of visual optic flow inputs during the tri-modal W-F-T condition did not exaggerate this change. Similarly, for the P3, condition impacted both amplitude and latency. Amplitudes were stronger in the S-NF-T condition compared to the two others, indicating that walking attenuated this peak, but adding flow in addition to walking was either not impactful enough to further attenuate the peak, or the attenuation between S-NF-T and W-NF-T was maximal, and indicative of a ceiling effect on the degree of attenuation possible. These effects were independent of group and response type.

A group level difference emerged regarding the inhibitory control implementation component, P3, which has previously been shown to be atypical in ASD [91, 128]. Here, P3 amplitudes during CRs were stronger in the TD group than the ASD group, regardless of experimental condition. In ASD, attenuated P3 amplitudes have been linked to difficulty inhibiting irrelevant stimuli and distractors [110, 129], and atypical stimulus processing [130]. Other studies have shown non-significant differences between ASD and TD groups’ P3 amplitudes [90, 91]. These prior studies vary in exact age ranges and experimental details, making it difficult to use their findings to interpret our results. The P3 has been shown to be reduced with increasing task difficulty [131], linked to inhibitory control deficits [132], and to involve both the cognitive and motor facets of successful inhibtion [133]. Our scalp topographies and statistical cluster plots support the interpretation that the ASD group had weaker activation related to this component during CRs, as evidenced by the significantly weaker activity in the P3’s corresponding time window and central-parietal scalp locations across all three task-inclusive experimental conditions. This is further corroborated by the poorer overall accuracy in the ASD group. This all suggests that the ASD group, regardless of motion state or optical flow presence, demonstrated difficulty with inhibitory control implementation. The lack of group differences at the N2 in this study indicates the groups were not different in the extent of their activation when processing inhibitory conflict, between Go and NoGo trials, though. When we compared the magnitude of the difference between Hits and CRs within each group with statistical cluster plots (Figs. 5, 6 and 7), strong differences between the groups were only robustly present in S-NF-T (Fig. 5b) condition. Positive t-value clusters (warm colors) indicated the inhibitory difference in the TD group is greater compared to ASD. Negative t-value clusters (cool colors) indicate the opposite. This indicates that addition of motor and sensory demands drove the groups to be more similar in the extent of the difference in neurophysiological responses to inhibitory conflict, not exaggerating the group differences, as we expected.

This is an analysis of a rich dataset yielding a complex set of exploratory results. Our original research questions, centered on the group differences between ASD and TD groups to modulate gait, behavior, and neurophysiology in response to single-, dual- and tri-modal demands. Doing the task while walking made all participants have less variable gait characteristics compared to walking while not doing the task. Optical flow did similarly. These adjustments are indicative of more stable gaits with better balance support, suggesting automatic and controlled walking when extra demands are put on the cognitive system. Walking, but only with flow, increased reaction times for all participants. However, only the TD group accompanied these reaction time adjustments with commensurate increases in cognitive task accuracy, though this was independent of flow. We found that the group with ASD demonstrated a walking-based maintenance to task performance accuracy as their TD counterparts, despite also having slowed reaction times when walking. Additionally, there was a modulation of the N2 and P3 peaks when participants were walking but this did not extend when optic flow was added. Similarly, this main effect across all participants is differentially related to behavior at the group level with TD showing improved accuracy but ASD showing no change. Our group inhibitory effect statistical cluster plots (Figs. 5, 6 and 7) also support this. The strongest group differences in the magnitude of the inhibitory effect (Hits vs. CRs) is during the S-NF-T condition. The small number of statistical clusters in the W-NF-T and W-F-T conditions shows that under dual- and tri-modal conditions, the groups reallocate neural activity similarly but, again, with differentiating impacts on task accuracy.

When walking, these two groups of adolescents made similar or the same adjustments to reaction time, gait parameters, and overall neurophysiology but resulting in different cognitive performance outcomes. The ASD group’s accuracy maintenance may be rooted in an overall inhibitory control impairment, as evidenced by many previous studies and our current group effect of accuracy. Neurophysiologically, this is supported by reduced P3 amplitudes during CRs in the ASD group across conditions, reflecting difficulty with the control implementation stage in evaluating and executing the requisite processes for the vGNG task. Or, the ASD group may have prioritized strategy maintenance over shifting, whereas the TD group chose to shift to adopting a speed-accuracy tradeoff when walking.

**Table 5:** Summary table of results. Main and interaction effect *p*-values from all repeated measure ANOVAs reported and Greenhouse-Geisser corrected. See results sections respective to each metric for details on *post-hoc* pairwise comparisons

	Condition (S-NF-T vs. W-NF-T vs. W-fFT)		Response Type (Hits vs. CRs)	Group (TD vs. ASD)	Condition * Response Type	Group * Response Type	Group * Condition
Visual Go-NoGo Cognitive Task Performance							
Accuracy (d’)	-			0.033	0.012		
Reaction Time	0.003			-	-		
Neurophysiology - ERPs							
P2 amplitude	-		< 0.001 (FCz)	-	-	-	-
N2 amplitude	0.014 (FCz)		-	-	0.039 (FCz)	-	-
P3 amplitude	< 0.001 (CPz)		< 0.001 (CPz)	-	-	0.006 (CPz)	-
P2 Latency	-		0.006 (FCz)	-	-	-	0.027 (FCz)
N2 Latency	-		< 0.001 (FCz),	-	-	-	-
P3 Latency	0.043 (CPz)		< 0.001 (CPz)	-	-	-	-
Gait Kinematics							
	Flow vs. No Flow	Task vs. No Task			Flow * Task		
Step Width	< 0.001	-		0.003	-		
Stride Time	0.022	-		-	-		
Stride Length	0.039	0.011		-	0.016		
CV% Step Width	0.019	< 0.001		-	-		
CV % Stride Time	-	< 0.001		0.014	-		
CV% Stride Length	-	< 0.001		0.019	-		

### Limitations

There are limitations to this study. Both groups had more males than females, with the ASD group heavily leaning male. This may have impacted the results, but research using an appropriate number of each sex is needed to confirm this. As mentioned earlier, the absence of a Standing-Flow-Task (S-F-T) condition does limit the flexibility of the statistical approaches we could take, thus increasing the possibility of errors due to the use of less stringent tests. Further, the current study does not have the complete data or statistical power needed to introduce assessment measures of characteristics of ASD, motor skills, or executive functions into our statistical models. Therefore, we emphasize that the results here are especially useful for future hypothesis generation to further explore what individual and/or group factors best explain the differences seen here.

Of note when contextualizing the gait measures is the group difference in chosen walking speed on the treadmill, with ASD participants choosing to walk at slower speeds. Previous research from the lab using a very similar experimental design compared EEG measures when sitting, walking deliberately (2.4 km/hour), and walking briskly (5.0 km/hour). No differences between the two different walking speed conditions were found. The participants in the current study chose speeds slower than that De Sanctis, et al. study, though their cohort consisted of neurotypical young adults [4]. Participants choosing their own walking speed on the treadmill for the experiment also introduces variability and subjectivity to the experiment. Most participants selected a speed slower than average adult walking speed, about 1.46 [134] to 1.12 [135] m/s for adults, and 1.1 m/s for young teenagers [136] (though literature for children is very limited). It is possible that this prevented the motor domain from fully asserting a true ‘demand’ on the overall system. However, both neurophysiology and cognitive performance measures were impacted by walking, indicating that even a casual walk at these speeds impacts the interplay between the three domains in our group and age range. Our participants’ speeds were comparable to that of previous MoBI and mobile EEG studies [98, 137, 138]. 

Further, walking on a treadmill creates a motor motivator that is external, rather than internal, which has been shown to differ from over-ground walking, [124, 139] and may differentially impact cognitive performance [140]. Despite this, individual participant choice was a practical approach given the age, height and weight, and motor capability range of the participants included in this study. Additionally, the logistics of the EEG equipment used here and other MoBI studies prohibit over-ground walking because the devices are anchored in the recording space. Some studies have used various mobile EEG and/or fNIRS hardware to accomplish over-ground experimental designs [126] (see Richer, Bradford, & Ferris, 2024 for a review). Additionally, consistent speeds via the treadmill create a consistent motor demand throughout all experimental blocks [4, 30]. Future studies should carefully consider the advantages and disadvantages respective to over-ground or treadmill walking when designing new experiments.

Another potential limitation of this study is the use of standing as a control for walking. Standing is not a load-less motor condition, and involves basic gross motor and balance control. It was recently shown that healthy young adults are unlikely to suffer detrimental cognitive or attention effects on the Stroop task between sitting and standing [141]. However, individuals with ASD have demonstrated differences in studies on standing postural control and stability [42, 52, 53, 68, 142]. Future studies within this and other populations with known motor control differences could consider including a seated condition as a more robust control.

Additionally, the participants here were adolescents and young adults covering an age range from 13 to 23 years. This covers a period of high changes in development. It is possible effects of different domains were shrouded by the large age range. Future work should aim to stratify by smaller age groups to fully understand if and how the developmental trajectory of these effects presents. Additionally, ASD is far from the only neurodevelopmental disorder with known dual-task and motor inhibition differences. There is vast potential for future research to explore other such groups using similar approaches, such as ADHD, head and brain injuries, and even Schizophrenia Spectrum Disorder.

These results are from a sample of individuals with expressive language and without intellectual impairment. Future work will be needed to test if these findings generalize to a broader range of individuals with an autism spectrum diagnosis, including those with more limited language and with intellectual impairment. This may require modification of methodological approaches, so that they are suitable for individuals with different support needs than our current sample.

Finally, this study had moderate sample sizes limited by some measures missing data points. Thus, several effects across variables that approached significance may have been under-powered. Some significant effects across variables did not maintain significance in *post-hoc* analyses. Other effects were significant, but with weak effect sizes which emphasizes the need for conservative interpretations. This all supports the need for follow-up hypothesis-driven studies.

## Conclusion

These data suggest that the dynamic interplay of motor, cognitive, and sensory processing modulates differently in adolescents with ASD, compared to those without. The same or similar modulations made to reaction time, gait, and neurophysiology in both groups led to accuracy maintenance for the ASD group but improvement for the TD group. The central remaining question is whether these results are indicative of atypical integration of these three domains in ASD, or a multi-modal interference resistance in the TD group, or something else.

All of this work provides support for further investigation via MoBI to help establish markers of discriminating between neurotypical individuals and those with neurocognitive and/or behavioral deficits that any single method alone may not be sensitive enough to detect. Further, utilization of more realistic experimental designs lends itself to a multi-faceted understanding of effects of cognitive-motor-sensory interactions in various populations and could become a powerful non-invasive tool to inform personalized approaches for developmental conditions such as ASD, thus making diagnosis more precise and treatment more effective and providing a more all-inclusive way to identify deficits and monitor treatment and therapy impacts.

## Supplementary Information

Supplementary Material 1.

## Data Availability

The senior authors (SM and JJF) will make data available to interested academic partners upon reasonable request.

## Abbreviations

ASD: Autism Spectrum Disorder
C-M: Cognitive-motor
CR: Correct rejection
CV%: Coefficient of variation
DT: Dual-tasking
EEG: Electroencephalography
ERP: Event Related Potential
fNIRS: Functional near-infrared spectroscopy
MoBI: Mobile Brain-Body Imaging
S-NF-T: Standing No-Flow Task
ST: Single-tasking
TD: Typically Developing
vGNG: Visual Go NoGo
W-F-T: Walking Flow Task
W-F-NT: Walking Flow No-Task
W-NF-T: Walking No-Flow Task
W-NF-NT: Walking No-Flow No-Task
