# Supplementary material for: A symphony of functioning: exploring the interplay of cognition, movement, and visual demands in adolescents on the autism spectrum using mobile brain-body imaging (MoBI)
Source: J Neurodev Disord. 2026 Feb 10;18:13. doi: 10.1186/s11689-026-09677-1 (PMC12990423; doi:10.1186/s11689-026-09677-1)
Supplement: Supplementary file 1 — Supplementary Material 1. [file 11689_2026_9677_MOESM1_ESM.docx]

Supplementary Material

Supplemental Table 1: The number of trials kept after all EEG preprocessing for each group within each condition and response type: **mean** (*std*).

| Condition & Response Type | Group | *n* | Mean | STD |
| --- | --- | --- | --- | --- |
| S_NF_T_Hit | TD | 18 | 333.22 | 89.706 |
|  | ASD | 20 | 337.85 | 63.129 |
| S_NF_T_CR | TD | 18 | 45.00 | 12.658 |
|  | ASD | 20 | 42.70 | 15.614 |
| W_NF_T_Hit | TD | 18 | 352.61 | 54.695 |
|  | ASD | 20 | 317.35 | 86.625 |
| W_NF_T_CR | TD | 18 | 51.83 | 13.682 |
|  | ASD | 20 | 41.15 | 15.288 |
| W_F_T_Hit | TD | 18 | 358.94 | 44.910 |
|  | ASD | 20 | 322.35 | 66.357 |
| W_F_T_CR | TD | 18 | 55.22 | 12.680 |
|  | ASD | 20 | 42.00 | 15.482 |
